# Interplay of genetic predisposition, plasma metabolome and Mediterranean diet in dementia risk and cognitive function

**DOI:** 10.1038/s41591-025-03891-5

**Published:** 2025-08-25

**Authors:** Yuxi Liu, Xiao Gu, Yanping Li, Fenglei Wang, Chirag M. Vyas, Cheng Peng, Danyue Dong, Yuhan Li, Yu Zhang, Yin Zhang, Oana A. Zeleznik, Jae H. Kang, Molin Wang, Frank B. Hu, Walter C. Willett, Olivia I. Okereke, A. Heather Eliassen, Peter Kraft, Meir J. Stampfer, Dong D. Wang

**Affiliations:** 1 Channing Division of Network Medicine, Department of Medicine, Brigham and Women’s Hospital and Harvard Medical School, Boston, MA USA; 2 Department of Epidemiology, Harvard T.H. Chan School of Public Health, Boston, MA USA; 3 Broad Institute of MIT and Harvard, Cambridge, MA USA; 4 Department of Nutrition, Harvard T.H. Chan School of Public Health, Boston, MA USA; 5 Department of Psychiatry, Massachusetts General Hospital and Harvard Medical School, Boston, MA USA; 6 Department of Biostatistics, Harvard T.H. Chan School of Public Health, Boston, MA USA; 7 Division of Cancer Epidemiology and Genetics, National Cancer Institute, Rockville, MD USA

**Keywords:** Alzheimer's disease, Genetic association study, Epidemiology, Predictive markers, Risk factors

## Abstract

Alzheimer’s disease (AD) and AD-related dementias (AD/ADRD) have a substantial genetic basis, with *APOE4* homozygotes increasingly recognized as a distinct genetic subtype. To identify genotype-specific metabolic pathways and modifiable risk factors, we integrated genetic, plasma metabolomic and dietary data from 4,215 women and 1,490 men in prospective cohorts. Here we show that the associations of 57 metabolites with dementia risk varied by *APOE4* genotype or other AD/ADRD risk variants. For example, cholesteryl esters and sphingomyelins were most strongly associated with increased dementia risk in *APOE4* homozygotes, whereas inverse associations with glycerides were specific to this genotype. Dimethylguanidino-valeric acid was more strongly associated with dementia risk among carriers of the rs2154481-C allele (*APP*). Adherence to the Mediterranean diet more effectively modulated dementia-related metabolites in *APOE4* homozygotes, suggesting targeted prevention strategies. Incorporating metabolomic data modestly improved dementia risk prediction, particularly during early follow-up. Mendelian randomization analysis identified 19 putative causal relationships between metabolites and cognitive outcomes, including protective effects of 4-guanidinobutanoate, carotenoids and *N*^6^-carbamoylthreonyladenosine. These findings reveal genotype-dependent metabolic profiles of cognitive health and support precision nutrition approaches for ADRD prevention.

## Abstract (web summary)

Genetic background, plasma metabolome and dietary data from 4,215 women and 1,490 men reveal 57 metabolites whose associations with dementia risk vary by *APOE4* genotype and other risk variants.

## Main

Alzheimer’s disease (AD) and AD-related dementias (AD/ADRD) are neurodegenerative disorders characterized by progressive decline in memory, cognitive function and the ability to perform daily activities^1^. AD/ADRD has a substantial genetic basis, with heritability estimated at up to 80% from twin studies^2^. The apolipoprotein E (*APOE*) gene is the strongest genetic risk factor for sporadic AD^3^; carrying 1 *APOE-ε4* (*APOE4*) allele increases risk 3–4-fold and 2 alleles increase risk 8–12-fold compared to the common *APOE-ε3* allele^4^. *APOE4* exacerbates amyloid-β (Aβ) pathology in the brain^5^ and is strongly linked to dysregulation in lipid metabolism and impaired cerebral glucose metabolism^6^, highlighting its multifaceted role in the pathology of ADRD. Recent studies further suggest that *APOE4* homozygotes exhibit unique clinical, pathological and biomarker changes that begin at younger ages^7^. Beyond *APOE*, genome-wide association studies (GWASs) have identified common genetic variants at >80 loci associated with AD/ADRD risk, including *ABCA7*, *BIN1* and *CR1*, confirming its polygenic basis and implicating key pathogenic pathways such as immune response and endocytosis^8,9^. However, previous research has predominantly focused on the genetic effects of a limited set of biomarkers that primarily capture Aβ and tau pathologies, often assessed during the prodromal stages of the disease or with limited duration of prospective follow-up. There is a notable lack of evidence about the impact of genetic factors on early stage biomarkers derived from high-throughput omics approaches, such as metabolomics, and their associations with ADRD risk. Furthermore, the potential to leverage identified gene–biomarker interactions to develop individualized prevention and treatment strategies remains largely unexplored.

Metabolomics provides a readout of the combined effects of genetic and environmental factors, offering an expansive snapshot of metabolic states^10^. Genetic variations, particularly in genes involved in enzyme and transporter functions, directly influence metabolite production, degradation and circulation^11^. Environmental factors, especially diet, interact with genetics to further shape the metabolome by introducing exogenous metabolites and modulating key metabolic processes, such as inflammation, energy production and oxidative stress^12,13^. Metabolomic profiles across various tissues, including plasma^14^, brain^15^ and cerebrospinal fluid^16^, have shown associations with ADRD risk and cognitive function. Emerging evidence suggests that the *APOE4* genotype may modulate associations between plasma metabolites and dementia risk^17–20^. For example, recent findings indicate that females carrying the *APOE4* allele have distinct metabolomic profiles, reflecting alterations in lipid and amino acid metabolism, potentially increasing susceptibility to AD^17,19^. Despite these findings, there is a lack of data from large prospective studies investigating the extent to which associations between the metabolome and cognitive outcomes vary by genetic background. In addition, it remains unclear whether certain modifiable risk factors, such as diet, can mitigate ADRD risk and cognitive decline by targeting specific metabolic pathways across different genetic risk groups.

To examine the interplay of genetics, the plasma metabolome and diet in relation to dementia risk and cognitive function, we conducted a prospective analysis of 4,215 women during a 34-year follow-up in the Nurses’ Health Study (NHS). Notably, we observed widespread variations in associations between metabolites and cognitive outcomes across genotypes, most notably among *APOE4* homozygotes. Associations of the Mediterranean diet (MedDiet) with metabolites and dementia risk were also genotype dependent, with metabolites mediating the MedDiet–dementia risk association only among *APOE4* carriers. Integration of genetics with metabolomics improved the prediction of cognitive outcomes. Key findings were replicated in 1,490 men in the Health Professionals Follow-Up Study (HPFS). Two-sample Mendelian randomization (MR) further supported causal relationships between plasma metabolites and cognitive outcomes. To our knowledge, this is one of the first studies to demonstrate genotype-dependent associations between metabolites and ADRD risk, with additional findings that inform individualized dietary approaches for ADRD prevention.

## Results

### Integrating genetics, plasma metabolomics and dietary intakes to study ADRD etiology in long-running prospective studies

**Fig. 1:**
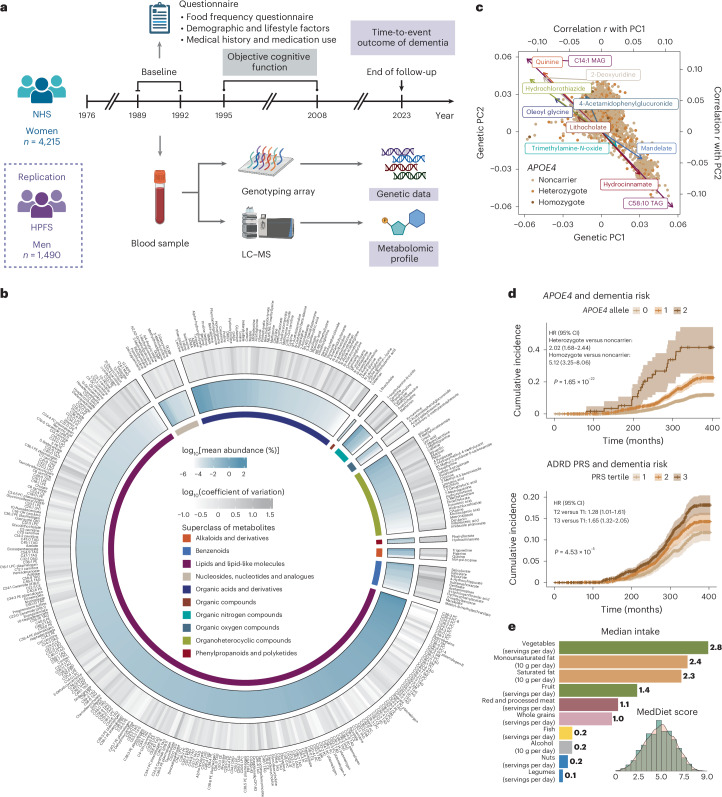
Prospective cohort studies examining the interrelationship of genetics, plasma metabolomics, MedDiet, cognitive function and dementia risk. **a**, Prospective follow-up of 4,215 women in the NHS from 1989 to 2023. Genetic and metabolomic profiles were generated from blood samples collected at baseline. Detailed demographic, lifestyle, dietary, medical history and medication use data were collected via questionnaires. Dementia cases were ascertained through the follow-up as a composite endpoint of incident dementia and death due to dementia. In addition, a telephone-based neuropsychological assessment battery was administered longitudinally from 1995 to 2008 to assess cognitive function in a subset of 1,037 participants. A total of 1,490 men from the HPFS were included as a replication cohort (Extended Data Fig. 1a). **b**, Distribution of plasma metabolites (*n* = 401). The outer circle represents the variation of each metabolite, with a gradient in gray indicating the coefficient of variation. The inner circle displays the mean relative abundance of each metabolite, shown as a gradient in blue. The innermost circle color codes represent the different HMDB superclasses defined based on chemical structural similarities. **c**, Overall genetic structure associated with individual metabolites. Each dot represents an individual and is colored by *APOE4* genotype, showing no clear pattern between the overall population substructure and *APOE4* genotype. The metabolites with the highest Pearson’s correlations with the top two genetic PCs from each metabolite superclass are included on the plot as arrows, colored by their superclass (see legend for **b**). The arrowhead coordinates represent the correlation coefficients of the metabolites with genetic PC1 and PC2. **d**, Associations between established genetic risk factors for AD/ADRD and dementia risk. The lines indicate cumulative incidence across *APOE4* genotypes and tertiles of the PRS of ADRD (excluding the *APOE* region) over the follow-up period, with shaded areas representing 95% CIs and *P* values from the log-rank test annotated. Consistent with the curves, unadjusted hazard ratios (HRs) were estimated using Cox proportional hazards (PH) model; covariate-adjusted HRs with 95% confidence intervals (CIs) are provided in Supplementary Table 4. Person time was accrued from baseline until the earliest occurrence of an incident dementia case, dementia death or the end of follow-up. No adjustment was made for multiple comparisons, because this was a hypothesis-driven analysis. **e**, A wide range of adherence to the MedDiet, as assessed by a dietary index and intake levels of food and nutrient components of MedDiet. All analyses and distributions were based on data from 4,215 NHS participants. All statistical tests were two sided. MAG, monoacylglycerol; TAG, triacylglycerol. Panel **a** created using BioRender.com. Source data

**Extended Data Fig. 1:**
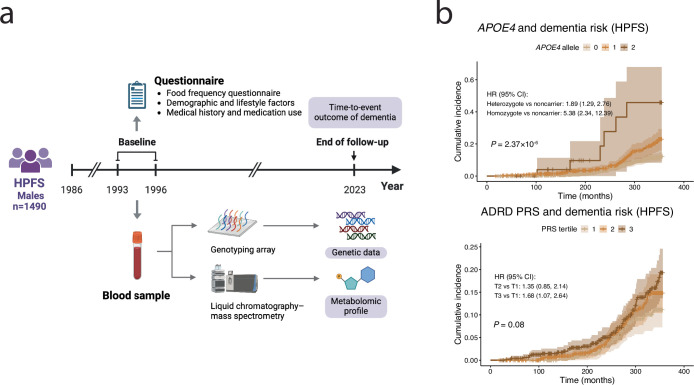
Overview of the Health Professionals Follow-Up Study as a replication cohort. (**a**) We prospectively followed 1,490 men in Health Professionals Follow-Up Study (HPFS) from 1993 through 2023. Genetic and metabolomic profiles were generated from blood samples collected at baseline. Detailed demographic, lifestyle, dietary, medical history and medication use data were collected via questionnaires. Dementia cases were ascertained through the follow-up as a composite endpoint of incident dementia and death due to dementia. (**b**) Associations between established genetic risk factors for Alzheimer’s disease and related dementias (ADRD) and dementia risk. Lines indicate cumulative incidence across *APOE4* genotypes and tertiles of polygenic risk score (PRS) of ADRD (excluding the *APOE* region) over the follow-up period, with shaded areas representing 95% confidence intervals (CIs) and *P* from the log-rank test annotated. Consistent with the curves, unadjusted hazard ratios (HRs) were estimated using Cox proportional hazards models; covariate-adjusted HRs with 95% CIs are provided in Supplementary Table 4. Person-time was accrued from baseline until the earliest occurrence of an incident dementia case, dementia death or the end of follow-up. No adjustment was made for multiple comparisons, as this was a hypothesis-driven analysis. The analyses were conducted among 1,490 HPFS participants. All statistical tests were two-sided. Panel **a** created using BioRender.com. Source data

We prospectively followed 4,215 women in the NHS from 1989 to 2023 (mean age, at baseline, 57 years; Fig. 1a and Supplementary Table 1), during which 485 participants developed dementia. In addition, we longitudinally assessed objective cognitive function using a telephone-based battery, including the Telephone Interview for Cognitive Status (TICS), in a subset of 1,037 participants (1995–2008). In the replication analyses in the HPFS, 1,490 men (mean age 63 years at baseline) were prospectively followed from 1993 to 2023, with 121 dementia cases documented (Extended Data Fig. 1a and Supplementary Table 2). Details of baseline characteristics in both cohorts are provided in Supplementary Text.

**Extended Data Fig. 2:**
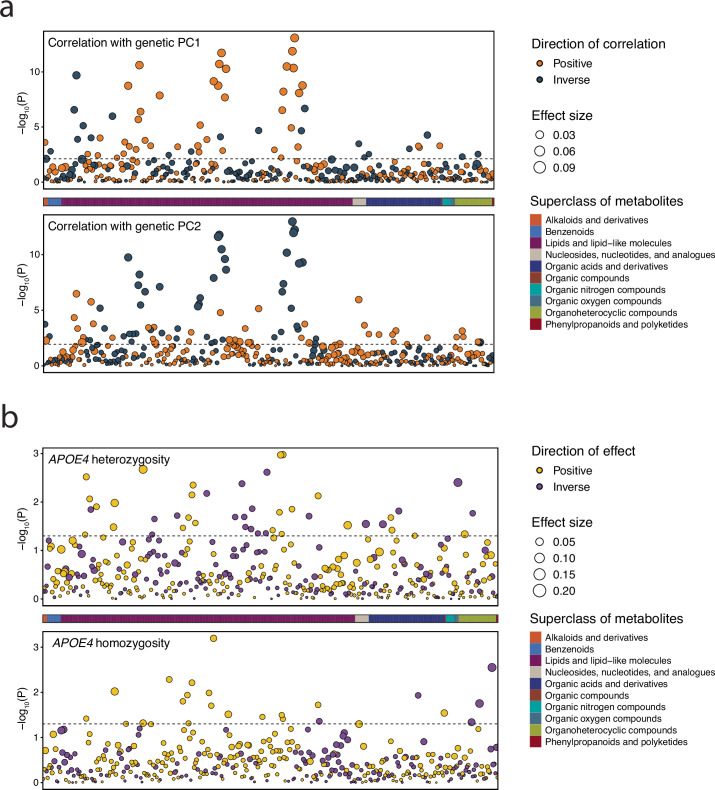
Associations between genetic factors and 401 metabolites in Nurses’ Health Study. (**a**) Correlations between genetic principal components (PCs) and metabolites: PC1 (top panel) and PC2 (bottom panel). The dashed lines denote false discovery rate (FDR) = 0.05, adjusted for multiple comparisons, based on Pearson’s correlation tests. (**b**) Associations of *APOE4* heterozygosity (top panel) and homozygosity (bottom panel) with metabolites, with *APOE4* noncarrier as the reference group from Cox proportional hazards models. Models were adjusted for age, the top 4 genetic PCs, and genotyping platforms. The dashed lines denote nominal *P* = 0.05 (none with FDR < 0.05). All analyses were conducted among 4,215 NHS participants. All statistical tests were two-sided. Source data

Metabolomic data were generated from plasma samples using a liquid chromatography–mass spectrometry (LC–MS)-based platform in both the NHS and the HPFS; a total of 401 metabolites from 10 Human Metabolome Database (HMDB) superclasses were included in the NHS analyses after quality control (QC), with 254 of the metabolites available in the HPFS (Fig. 1b and Supplementary Table 3). Genotyping data were generated from blood samples, followed by QC and imputation (Methods). We extracted the two *APOE* variants along with 73 other common variants identified from AD/ADRD GWASs^9,21^ and calculated two polygenic risk scores (PRSs) for ADRD; one included the two *APOE* variants and one excluded the *APOE* region, using weights from published studies^9,21^. We first investigated the global influence of genetic variations on the plasma metabolome, using principal components (PCs) to capture genetic structure. Strong correlations were observed between genetic PC1 or PC2 and metabolites previously linked to ADRD risk, such as trimethylamine *N*-oxide^22^ and lithocholate^23^ (Fig. 1c and Extended Data Fig. 2a). We next assessed the specific influence of *APOE4* genotype on the metabolome. As expected, *APOE4* homozygosity was broadly associated with elevated lipid metabolites compared to noncarriers (Extended Data Fig. 2b).

To validate the dementia outcome, we examined plasma phosphorylated tau 217 (p-tau217), an established biomarker for early AD diagnosis^24^, in 103 NHS participants and found an approximately 3-fold higher dementia risk comparing the highest and lowest quartiles of p-tau217. We further confirmed that carrying *APOE4* alleles or having a higher PRS of ADRD was associated with significantly increased risk of dementia and poorer cognitive function in both cohorts (Fig. 1d, Extended Data Fig. 1b, Supplementary Fig. 1, Supplementary Table 4 and Supplementary Text).

We collected long-term dietary data using extensively validated semiquantitative food frequency questionnaires (SFFQs) in both cohorts. To assess dietary quality, we employed the MedDiet index, the only dietary pattern causally linked to delayed cognitive decline in a long-term, randomized controlled trial^25^. A widespread distribution of MedDiet adherence was observed (Fig. 1e), with higher MedDiet index scores associated with older age, lower body mass index, higher education level and more physical activity (Supplementary Tables 2 and 5).

### *APOE4* homozygosity exhibited distinct metabolomic profiles of dementia risk

**Fig. 2:**
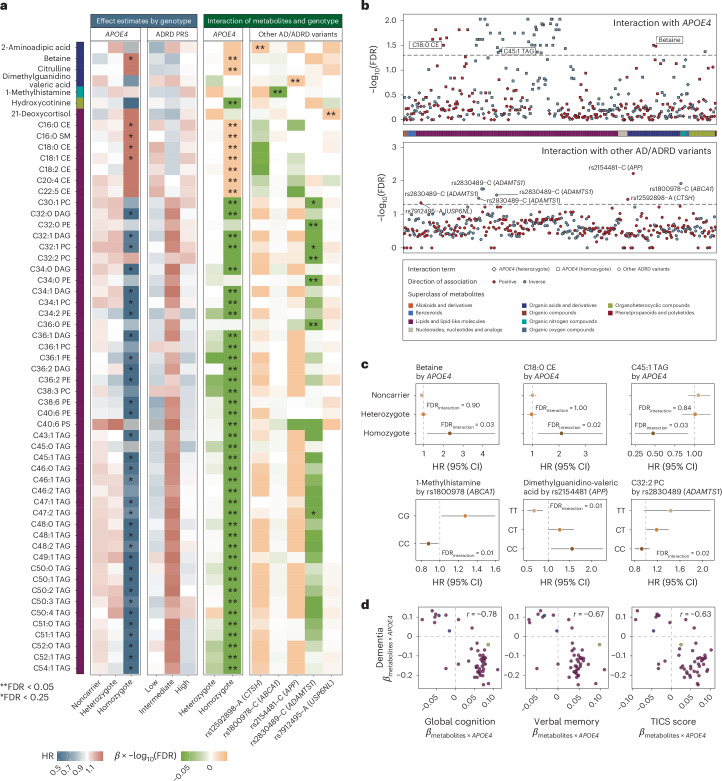
Associations of plasma metabolomic profiles, dementia risk and cognitive function differ according to individuals’ genetic predisposition to AD/ADRD. **a**, Significant variation in the association between metabolites and dementia risk across different genotypes. Left, in the two heatmaps, the color gradient denoting the HR for dementia risk per 1-s.d. increment in metabolite levels among individuals with different genetic predispositions, as defined by *APOE4* genotype or PRS of ADRD (including the *APOE* variants), estimated using Cox PH model. Only metabolites with FDR < 0.05 for their interaction terms with genotype are displayed in the heatmaps. Right, the color gradient in the two heatmaps representing the product of the *β* coefficient and the −log_10_(FDR) of the interaction term between each metabolite and genotype, as defined by the *APOE4* genotype or other common AD/ADRD genetic variants, from Cox PH model. In all heatmaps, associations or interactions with FDR < 0.05 are indicated by double asterisks (**) and those with FDR < 0.25 by a single asterisk (*). Metabolites are grouped according to HMDB superclasses. The analyses were conducted among 4,215 NHS participants. **b**, Gene–metabolite interactions related to dementia risk widely distributed across metabolite superclasses and genotypes. The Manhattan plot displays metabolome-wide interaction results, represented by −log_10_(FDR) values for interaction terms from Cox PH models. Each dot represents a metabolite colored by the direction of interaction and grouped by HMDB superclass. Top, for the *APOE4* genotype, the data point with the lower FDR between heterozygous (diamond) and homozygous (square) *APOE4* interactions included for each metabolite. Bottom, for other common AD/ADRD variants, the most significant interaction across all 73 variants shown for each metabolite. The analyses were conducted among 4,215 NHS participants. **c**, Selected associations between metabolites and dementia risk with FDR for interaction <0.05, stratified by genotype. The first row presents stratified HRs and 95% CIs for dementia risk per 1-s.d. increment in metabolite level, categorized by the *APOE4* genotype, with FDR values for interaction terms between *APOE4* heterozygosity and homozygosity annotated (using the noncarrier as the reference group). The second row displays stratified results (HR and 95% CIs per 1-s.d. increment in metabolite level) by AD/ADRD variants, with FDR values for interaction terms with the variant effect allele dosage annotated. Genotype groups were defined based on rounded allele dosages. Results for the rs1800978 GG genotype group are excluded due to data sparsity. The analyses were conducted across 4,215 NHS participants. **d**, Consistency of metabolite–*APOE4* interaction results across models with dementia risk and cognitive function as dependent variables. Each dot represents a metabolite with significant *APOE4* interactions, colored by the HMDB superclass. Pearson’s correlation coefficients in the *β* coefficients for interaction terms between metabolites and *APOE4* carrier status estimated from Cox PH models, with dementia as the dependent variable, and from generalized linear models, with cognitive function scores as the dependent variable, are annotated on each figure. *APOE4* carriers were not further divided into heterozygotes and homozygotes due to data sparsity among homozygotes with non-missing values for each metabolite in the cognitive function subset. Dementia risk analyses were conducted among 4,215 NHS participants and cognitive function analyses among 1,037 NHS participants. All statistical tests were two sided. DAG, diacylglycerol; PC, phosphatidylcholine; PE, phosphatidylethanolamine; TAG, triacylglycerol. Source data

**Extended Data Fig. 3:**
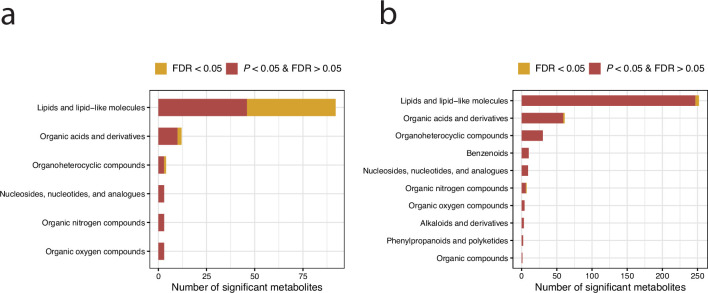
Number of metabolites with significant interactions with genetic factors in relation to dementia risk in Nurses’ Health Study by Human Metabolome Database superclass. (**a**) Number of metabolites with significant interactions with *APOE4* genotype. (**b**) Number of metabolites with significant interactions with other common AD/ADRD variants. Bars indicate the number of associations that reached nominal significance (*P* < 0.05) based on the Cox proportional hazards models, and those that remained significant after adjustment for multiple comparisons (false discovery rate <0.05). All statistical tests were two-sided. Source data

We identified 49 significant interactions of metabolites with *APOE4* genotypes in relation to dementia risk at a false discovery rate (FDR) < 0.05 (Fig. 2a,b, Extended Data Fig. 3 and Supplementary Table 6). All significant interactions were specific to *APOE4* homozygotes, aligning with recent findings on this isoform in AD pathology^7^ and suggesting that it exhibits a distinct plasma metabolomic profile associated with ADRD risk, evident even decades before disease onset.

Subgroup analyses further confirmed distinct association patterns between metabolites and dementia risk among *APOE4* homozygotes compared to others, with variations in both the magnitude and, in some cases, the direction of associations (Fig. 2a, Supplementary Fig. 2 and Supplementary Table 7). For example, a significant positive association between betaine and dementia risk was observed only among *APOE4* homozygotes (Fig. 2c). Plasma betaine levels reflect both dietary intake and one-carbon metabolism activity^26^. The protective, albeit nonsignificant, association observed among *APOE4* noncarriers and heterozygotes may primarily reflect dietary betaine’s benefit for cognitive health^27^, whereas, in *APOE4* homozygotes, elevated betaine may indicate methylation imbalance and metabolic dysregulation, contributing to an increased dementia risk^26,28^. We observed broadly positive associations across cholesteryl esters (CEs), sphingomyelins (SMs) and dementia risk in all genetic risk groups, with the strongest associations in *APOE4* homozygotes. The *APOE4* allele promotes the accumulation of cholesterol and CEs in the brain^29^, activating inflammatory pathways that exacerbate neuronal damage and contributing to the formation of amyloid plaques and tau tangles, thereby increasing dementia risk^30^. We observed inverse associations between glycerides and dementia risk, specifically among *APOE4* homozygotes, where elevated glyceride levels likely reflect this reduced delipidation, which may, in turn, limit the aggregation of apolipoprotein E (ApoE) and the formation of amyloid plaques^31^.

**Extended Data Fig. 4:**
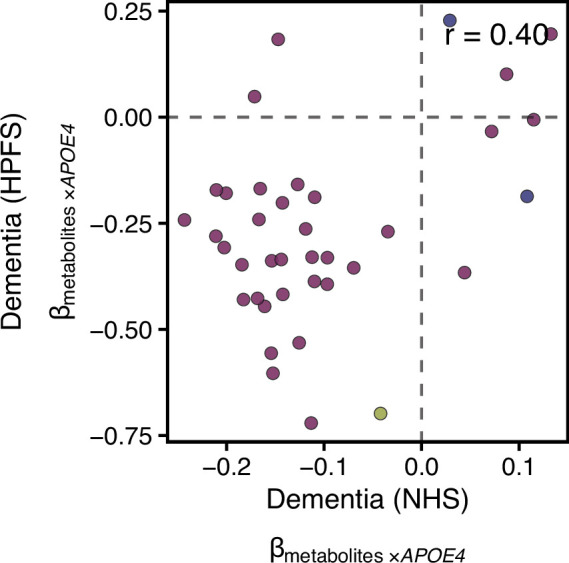
Consistency of metabolite-*APOE4* interactions in Nurses’ Health Study and Health Professionals Follow-Up Study. Each dot represents a metabolite with significant *APOE4* interactions in Nurses’ Health Study (NHS; n = 38 out of 49 available in Health Professionals Follow-Up Study [HPFS]), colored by the Human Metabolome Database superclass (see Fig. 2 for legend). Pearson’s correlation coefficients in the *β* coefficients for interaction terms between metabolites and *APOE4* carrier status estimated from Cox proportional hazards models with dementia as the dependent variable in NHS and HPFS is annotated on the figure. *APOE4* carriers were not further stratified into heterozygotes and homozygotes due to data sparsity among homozygotes with non-missing values for each metabolite in HPFS. Analyses were conducted among 4,215 NHS and 1,490 HPFS participants. All statistical tests were two-sided. Source data

Replication of the findings for dementia risk in objective cognitive function yielded broadly consistent results (Pearson’s *r* ranging from −0.78 to −0.63 for the interaction effect estimates; Fig. 2d and Supplementary Tables 8 and 9). Sensitivity analyses, excluding family history of dementia from the covariates or modeling dementia case and death separately, showed similar results (Supplementary Figs. 3 and 4). Independent replication of the NHS findings in the HPFS yielded broadly consistent results (Pearson’s *r* = 0.40), with 32 out of 38 significant interactions (84.2%) in the same direction. Notably, 4 interactions reached *P* < 0.05 and 10 reached *P* < 0.10, including inverse interactions with glycerides, although some variation between women and men remained (Extended Data Fig. 4 and Supplementary Table 10).

### Common AD/ADRD risk variants modified the associations between plasma metabolites and dementia risk

Although *APOE4* is the major contributor to the genetic risk of dementia, recent GWASs have identified many other common variants linked to AD/ADRD risk^8,9^. We examined how these risk variants, either aggregated into PRSs or as individual variants, may modify the associations between metabolites and dementia risk (Supplementary Tables 11 and 12). Although no significant interaction between PRSs and metabolites was detected after multiple testing correction (Fig. 2a and Supplementary Tables 13 and 14), we identified eight significant interactions between individual AD/ADRD variants, including those mapped to *ABCA1*, *APP*, *ADAMTS1*, *CTSH* and *USP6NL*, and metabolites in relation to dementia risk with an FDR < 0.05 (Fig. 2a,b, Supplementary Fig. 5 and Supplementary Table 15).

We observed that the positive association between 1-methylhistamine, a metabolite involved in immune and inflammatory responses in the brain^32^ (Fig. 2c), and dementia risk was significantly more pronounced in individuals carrying the rs1800978-G allele mapped to *ABCA1*, a gene that plays a crucial role in clearing Aβ peptide from the brain^33^. Dimethylguanidino-valeric acid, a metabolite associated with impaired fatty acid and amino acid metabolism, showed a stronger positive association with dementia risk among individuals carrying the C allele at rs2154481, a variant mapped to the *APP* gene that encodes Aβ precursor protein (APP), suggesting that dysregulated lipid and glucose metabolism may influence the processing of APP, leading to increased Aβ production^34^. In addition, lipid metabolites showed significant interactions with AD/ADRD genetic variants. For example, C32:2 phosphatidylcholine exhibited a positive interaction with an *ADAMTS1*-linked variant (rs2830489-T), implicating extracellular matrix remodeling and neuroinflammation in neurodegeneration^35^ (Supplementary Text).

### MedDiet may more effectively modulate metabolites implicated in dementia risk in *APOE4* homozygotes

A key distinction between metabolomics and genetics is that metabolites can be modified by exogenous factors and may serve as targets for intervention; in particular, diet significantly impacts the metabolome^36^. We thus examined whether diet, specifically the MedDiet, which has been implicated in cognitive health^25^, could modulate metabolite levels in individuals with different genetic predispositions to AD/ADRD.

**Fig. 3:**
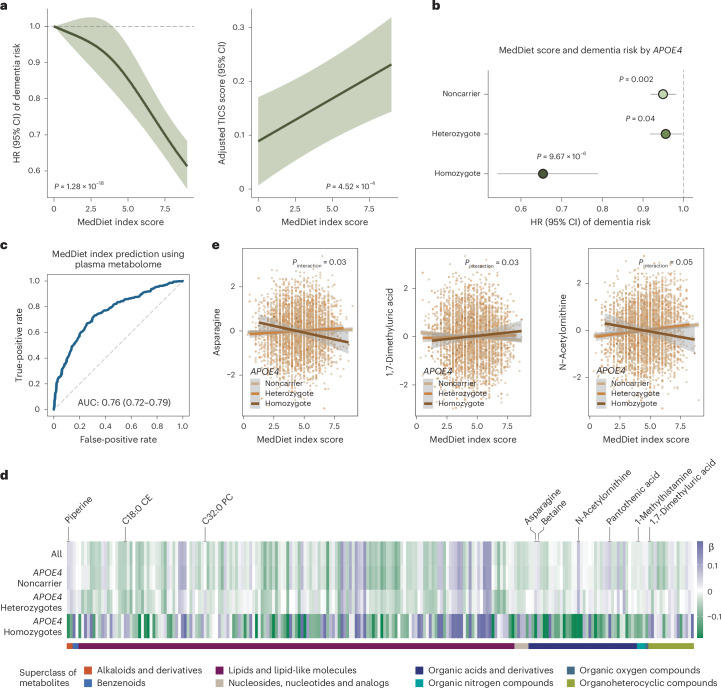
MedDiet adherence is associated with cognitive health and plasma metabolites in an *APOE4*-dependent manner. **a**, Higher adherence to the MedDiet prospectively associated with a lower risk of dementia and enhanced cognitive performance, as assessed by the telephone-based neuropsychological assessment battery (TICS). For dementia risk analysis, a restricted cubic spline Cox PH model estimated HRs and 95% CIs across varying levels of the MedDiet index, using 0 as the reference. The *P* value from a likelihood ratio test comparing the model without the MedDiet index and the model with its spline term is annotated. For the TICS score analysis, a generalized linear model estimated the adjusted TICS score and corresponding 95% CI across MedDiet index levels, with *P* values annotated. The analyses were conducted among NHS participants with cognitive and dietary data (*n* = 86,740 for dementia analysis and *n* = 16,244 for cognitive function analysis). **b**, The protective association between adherence to the MedDiet and risk of dementia most pronounced among *APOE4* homozygotes. Stratified HR and 95% CIs for dementia risk per a 1-unit increment in the MedDiet index score, categorized by the *APOE4* genotype, were estimated from Cox PH models, with stratified *P* values annotated (unadjusted for multiple comparisons in the hypothesis-driven analysis). The analyses were conducted among NHS participants with genetic, dietary and dementia outcome data (*n* = 16,497). **c**, Strong association between adherence to MedDiet and the overall plasma metabolome from an RF model to classify individuals in the top versus the bottom quartile of the MedDiet index based on plasma metabolites. For the RF classification, the dataset was randomly divided into training (60%) and test (40%) sets. The ROC curve for the test set is shown, with the AUC and 95% CI annotated on the plot. The analyses were conducted among 4,215 NHS participants. **d**, Associations between MedDiet adherence and plasma metabolite levels differing by *APOE4* genotype. The heatmap shows *β* coefficients representing a 1-s.d. increment in the MedDiet index from a generalized linear model, with plasma metabolite levels as the dependent variable. The analyses were conducted among 4,215 NHS participants. **e**, Select associations between the MedDiet index and plasma metabolite levels with *P* < 0.05 from the likelihood ratio test for the interaction between *APOE4* genotype and MedDiet index in relation to metabolites, using a generalized linear model stratified by *APOE4* genotype. Covariate-adjusted residuals of metabolites are shown along with fitted linear regression lines, 95% CIs and *P* values for interaction. These results were not adjusted for multiple testing. The analyses were conducted across 4,215 NHS participants. All statistical tests were two sided. Source data

**Extended Data Fig. 5:**
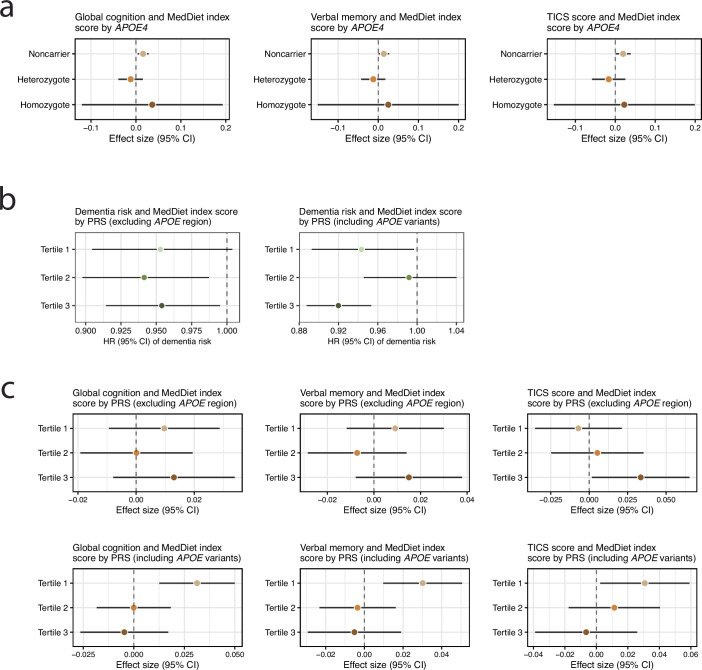
Associations of Mediterranean diet index score with dementia risk and cognitive function by genetic factors in Nurses’ Health Study. (**a**) Associations between Mediterranean diet (MedDiet) index score and cognitive function scores by *APOE4* genotype from generalized linear models. (**b**) Associations between MedDiet index score and dementia risk by tertiles of ADRD PRSs from Cox proportional hazards models. (**c**) Associations between MedDiet index score and cognitive function scores by tertiles of ADRD PRSs from generalized linear models. Stratified *β* coefficients (for cognitive function) and hazard ratios (HRs; for dementia risk), along with 95% confidence intervals (CIs) per one-unit increase in the MedDiet index score, are shown by *APOE4* genotype or PRS categories. The analyses were conducted among NHS participants with genetic, dietary, and cognitive outcome data (n = 16,497 for dementia analyses and n = 3,770 for cognitive function analyses). Source data

**Extended Data Fig. 6:**
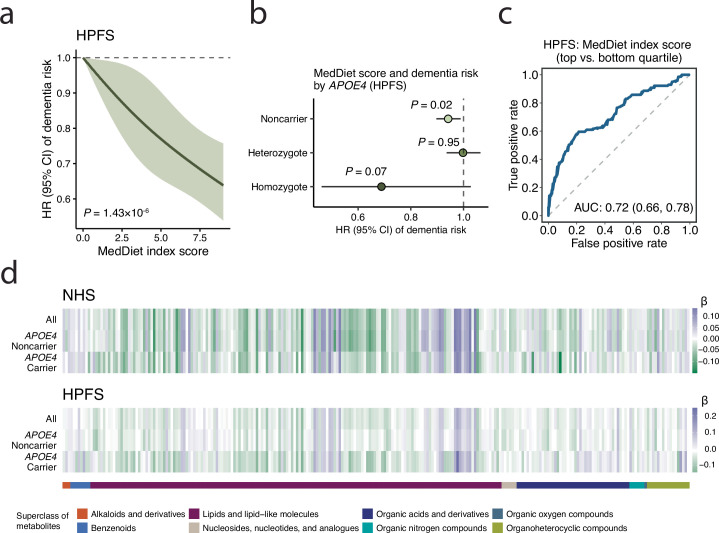
Replication of the associations of Mediterranean diet index score with plasma metabolites and dementia risk by *APOE4* genotype in Health Professionals Follow-Up Study. (**a**) Higher adherence to the Mediterranean diet (MedDiet) was prospectively associated with a lower risk of dementia. A restricted cubic spline Cox proportional hazards (PH) model estimated hazard ratios (HRs) and 95% confidence intervals (CIs) across varying levels of the MedDiet index. The *P* value from the likelihood ratio test comparing the model without the MedDiet index and the model with its spline term is annotated. This analysis was conducted among Health Professionals Follow-Up Study (HPFS) participants with dementia and dietary data (n = 43,500). (**b**) The protective association between adherence to the MedDiet and risk of dementia was most pronounced among *APOE4* homozygotes. Stratified HR and 95% CIs for dementia risk per one-unit increment in the MedDiet index score, categorized by *APOE4* genotype, were estimated from Cox PH models, with stratified *P* annotated (unadjusted for multiple comparisons in the hypothesis-driven analysis). The analyses were conducted among HPFS participants with genetic, dietary, and dementia outcome data (n = 9,828). (**c**) Strong association between adherence to MedDiet and the overall plasma metabolome profile identified by a random forest (RF) model to classify individuals in the top versus bottom quartile of the MedDiet index based on plasma metabolites. For the RF classification, the dataset was randomly divided into training (60%) and test (40%) sets. The receiver operating characteristic curve for the test set is shown, with the area under the curve (AUC) and 95% CI annotated on the plot. The analyses were conducted among 1,490 HPFS participants. (**d**) Associations between MedDiet adherence and plasma metabolite levels are consistent in NHS and HPFS and differ by *APOE4* genotype. The heatmap shows *β* coefficients representing a 1-s.d. increment in the MedDiet index from a generalized linear model, with plasma metabolite levels as the dependent variable, restricting to the 254 metabolites available in both cohorts. In the metabolite analysis, *APOE4* carriers were not further stratified into heterozygotes and homozygotes due to data sparsity among homozygotes with non-missing values for each metabolite in HPFS. The analyses were conducted among 4,215 NHS and 1,490 HPFS participants. All statistical tests were two-sided. Source data

**Extended Data Fig. 7:**
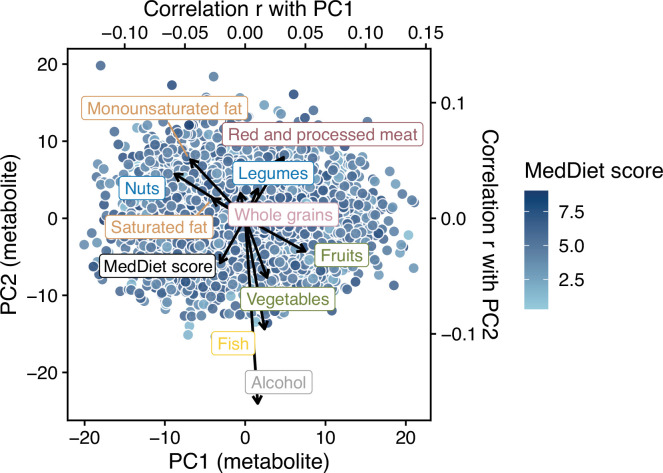
Correlation coefficients between principal components of metabolites and the Mediterranean diet index, along with its individual components in Nurses’ Health Study. Metabolite principal component (PC) plot with correlation coefficients between metabolite PC1 and PC2 and Mediterranean diet (MedDiet) score and its components. Each dot represents an individual and is colored by the MedDiet score. MedDiet score and its components are shown on the plot as arrows; the coordinates of the arrow heads represent their Pearson’s correlation coefficients with metabolite PC1 and PC2. The analyses were conducted among 4,215 participants in Nurses’ Health Study. Source data

We found that individuals with greater adherence to the MedDiet had a significantly lower risk of dementia and better cognitive function (Fig. 3a and Supplementary Fig. 6). Notably, these protective associations for dementia risk were more pronounced among *APOE4* homozygotes compared to noncarriers and heterozygotes (Fig. 3b), although no clear trend was observed when stratifying by ADRD PRS (Extended Data Fig. 5). The same patterns were observed in the HPFS (Extended Data Fig. 6a,b). Next, to evaluate MedDiet’s impact on the overall metabolomic profile, we used a random Forest (RF) classifier to distinguish individuals with high versus low MedDiet adherence based on plasma metabolite levels. This classifier demonstrated excellent performance, with an area under the receiver operating characteristic (ROC) curve (AUC) of 0.76 in the NHS (Fig. 3c) and 0.72 when replicating in the HPFS (Extended Data Fig. 6c). In addition, individual components of the MedDiet, such as nuts, fruit and monounsaturated fats, were strongly associated with overall metabolomic patterns, as captured by the top two metabolite PCs (Extended Data Fig. 7).

**Extended Data Fig. 8:**
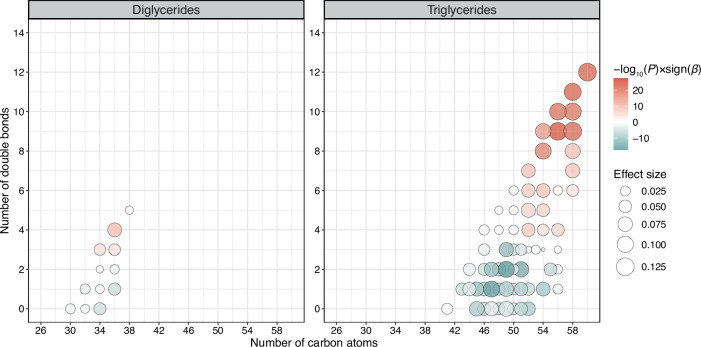
Associations of glycerides with the Mediterranean diet index in Nurses’ Health Study. Each dot represents a metabolite (diglycerides on the left panel and triglycerides on the right panel), color-coded by the direction and significance level of its association with the Mediterranean diet index. The size of each dot represents the effect size of the association. The *P* value is unadjusted for multiple comparisons given the exploratory nature of the analysis. The analyses were conducted among 4,215 NHS participants. All statistical tests were two-sided. Source data

To assess whether the metabolic response to MedDiet is genotype dependent, particularly in relation to *APOE4*, we examined the association between MedDiet adherence and metabolite levels across *APOE4* genotypes. As anticipated, the overall association patterns differed among *APOE4* homozygotes and heterozygotes compared to noncarriers (Fig. 3d and Supplementary Table 16), with interaction analysis supporting these distinctions (Supplementary Table 17). Similar patterns were observed in the HPFS (Extended Data Fig. 6d, Supplementary Fig. 7 and Supplementary Table 18). Consistent with findings from the PREDIMED trial involving a randomized MedDiet^37^, greater adherence to the MedDiet was associated with higher levels of unsaturated glycerides and lower levels of saturated glycerides, lipid patterns potentially beneficial for cognitive health, as well as increased levels of established neuroprotective compounds, including piperine, betaine and pantothenic acid^38,39^ (Extended Data Fig. 8 and Supplementary Table 16). Among the metabolites showing suggestive *APOE4*–MedDiet interactions (*P* < 0.05), we observed an inverse association between MedDiet and asparagine levels exclusively among *APOE4* homozygotes (Fig. 3e). This may reflect MedDiet-driven changes in amino acid metabolism, including glutaminolysis and the tricarboxylic acid cycle, which are key to asparagine regulation. Given asparagine’s role in protein and nucleotide synthesis, its reduction may signal broader metabolic benefits specific to *APOE4* homozygotes^40^. We also identified nominally significant interactions between MedDiet and the *APOE4* genotype in relation to 1,7-dimethyluric acid, a derivative of caffeine metabolism with established antioxidant properties and potential neuroprotective effects^41^ (Supplementary Text).

Furthermore, we found that 39.5% of the association between MedDiet adherence and dementia risk was mediated by a set of metabolites among *APOE4* carriers (*P* = 0.05), whereas no mediation effect was observed among noncarriers or in the full dataset (Methods). Besides the *APOE4* genotype, broad interactions were also observed for ADRD PRS and individual variants (Supplementary Tables 19–21). These findings collectively indicated that the MedDiet’s potential to modulate cognitive health-related metabolites varied by *APOE4* genotype, suggesting that this dietary pattern could be an effective strategy to delay dementia onset in *APOE4* homozygotes, despite their higher risk profile.

### Prediction of dementia risk using genetic, metabolomic and dietary factors

**Fig. 4:**
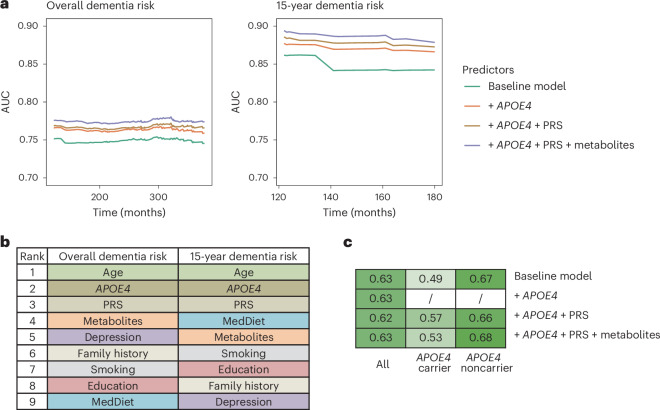
Integrating genetic variation with plasma metabolites and MedDiet enhances the prediction of dementia risk and cognitive status. **a**, The inclusion of genetic factors improving dementia risk prediction using Cox PH model, with an additional modest enhancement when plasma metabolites also included. Time-dependent ROC curve analyses were conducted for dementia risk over both the entire follow-up period and the first 15 years of follow-up. The baseline model predictors included age, family history of dementia, education level, smoking status, history of depression or regular antidepressant use and MedDiet index. The PRS of ADRD excluded variants in the *APOE* region (see Methods for selection of metabolite predictors). **b**, Plasma metabolites among the top contributors for predicting dementia risk as quantified by the SHAP value. Feature contributions were evaluated for Cox PH model to predict overall and 15-year dementia risk, including the full list of predictors. SHAP values were calculated for each category of predictors by summing the SHAP value of all predictors in that category. Features were ranked by the SHAP value from the highest to the lowest for predicting the overall and 15-year dementia risk. **c**, Integration of genetic and metabolomic data enhancing cognitive status prediction within *APOE4* subgroups. The heatmap displays AUCs from an RF model classifying participants in the highest versus the lowest tertile of the overall TICS score. In subgroup analyses by *APOE4* carrier status, *APOE4* genotype was excluded as a predictor. For all analyses, the NHS dataset (*n* = 4,215) was randomly divided into training (60%) and test (40%) sets; models were fitted on the training set and evaluated on the test set. All results shown are from the test set. Source data

**Extended Data Fig. 9:**
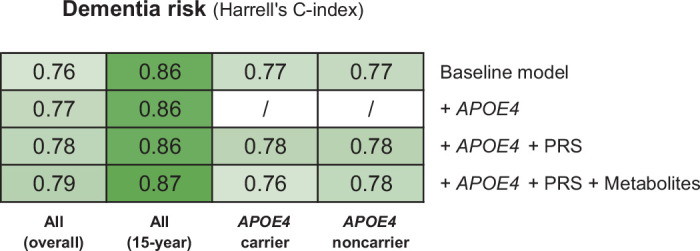
Harrell’s C index for predicting dementia risk from models with different predictors in Nurses’ Health Study. *APOE4* genotype was not included as a predictor in the subgroup analysis within *APOE4* carrier and noncarrier subgroups. The full NHS dataset (n = 4,215) was randomly split into training (60%) and test (40%) sets. All prediction models were fitted in the training set and were evaluated in the test set. All results shown are from the test set.

Given that genetics, metabolites and MedDiet adherence are all linked to dementia risk, we further examined how incorporating these factors could enhance the prediction of cognitive outcomes. Compared to a baseline model, which included age, family history of dementia, education level, smoking status, history of depression or regular antidepressant drug use and the MedDiet index, adding *APOE4* and ADRD PRS to Cox model moderately improved the performance for predicting dementia risk (Fig. 4a; average AUC improved from 0.75 to 0.77). This also demonstrated that genetic factors capture additional information beyond family history of dementia and that the ADRD PRS adds modest but incremental predictive value beyond *APOE4*. Adding metabolites predictive of dementia risk to the model further improved the time-specific model performance, indicating that metabolites provide additional predictive value beyond the MedDiet and other major dementia risk factors (average AUC = 0.78; Supplementary Table 22). As expected, these baseline characteristics were better at predicting short-term dementia risk, that is, 15-year risk, compared to long-term risk, which may be attributed to the inherent within-individual variability of metabolomic measurements over longer follow-up periods, potentially introducing random measurement error. Similar patterns were observed for Harrell’s C-index (Extended Data Fig. 9). The performance of different models in *APOE4* subgroups did not substantially deviate, likely due to the limited sample size and number of cases within these subgroups, which may have introduced substantial instability. Future studies with larger sample sizes are warranted to more reliably evaluate prediction performance within *APOE4* subgroups.

**Extended Data Fig. 10:**
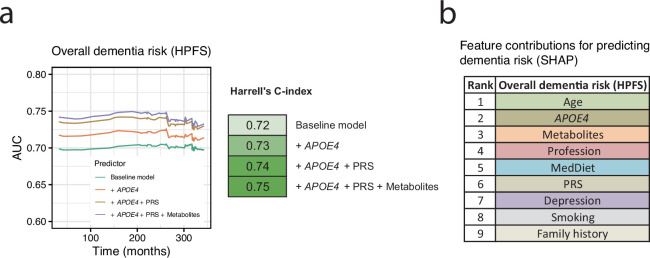
Integrating genetic variation with plasma metabolites and Mediterranean diet enhances the prediction of dementia risk in the Health Professionals Follow-Up Study. (**a**) The inclusion of genetic factors improved dementia risk prediction using Cox proportional hazards (PH) model, with an additional modest enhancement when plasma metabolites were also included. The baseline model predictors included age, family history of dementia, profession, smoking status, history of depression or regular antidepressant use, and Mediterranean diet (MedDiet) index. The polygenic risk score (PRS) of Alzheimer’s disease and related dementias excluded variants in the *APOE* region. (**b**) Plasma metabolites are among the top contributors for predicting dementia risk as quantified by the Shapley Additive Explanations (SHAP) value. Feature contributions were evaluated for the Cox PH model for predicting dementia risk including the full list of predictors. SHAP values were calculated for each category of predictors by summing the SHAP value of all predictors in that category. Features were ranked by the SHAP value from the highest to the lowest for predicting dementia risk. We did not assess 15-year dementia risk in the Health Professionals Follow-Up Study (HPFS) due to the limited number of dementia cases. For all analyses, the HPFS dataset (n = 1,490) was randomly divided into training (60%) and test (40%) sets; models were fitted on the training set and evaluated on the test set. All results shown are from the test set. Source data

We employed the SHapley Additive exPlanation (SHAP) values to quantify the contributions of individual predictors to dementia risk predictions. As anticipated, age, *APOE4* and ADRD PRS were among the top contributors (Fig. 4b and Supplementary Table 23). Plasma metabolites meaningfully contributed to the prediction throughout the entire follow-up period, with their overall contribution ranking just below age and genetic factors. In contrast, for short-term risk prediction, MedDiet emerged as a major predictor. The relatively modest contribution of MedDiet to overall follow-up predictions likely reflects the use of baseline dietary data only, which does not account for changes in dietary behavior over time. We observed similar patterns in the HPFS (Extended Data Fig. 10a,b) and when predicting a dichotomized TICS score (highest versus lowest tertile) within *APOE4* subgroups, whereas no improvement was observed in the full dataset (Fig. 4c and Supplementary Fig. 8). The unstable results for cognitive score prediction likely reflect limited sample size in the subset. Larger studies are needed to further assess the predictive utility of these models for cognitive function.

### Putative causal relationships between metabolomic features and cognitive outcomes

**Fig. 5:**
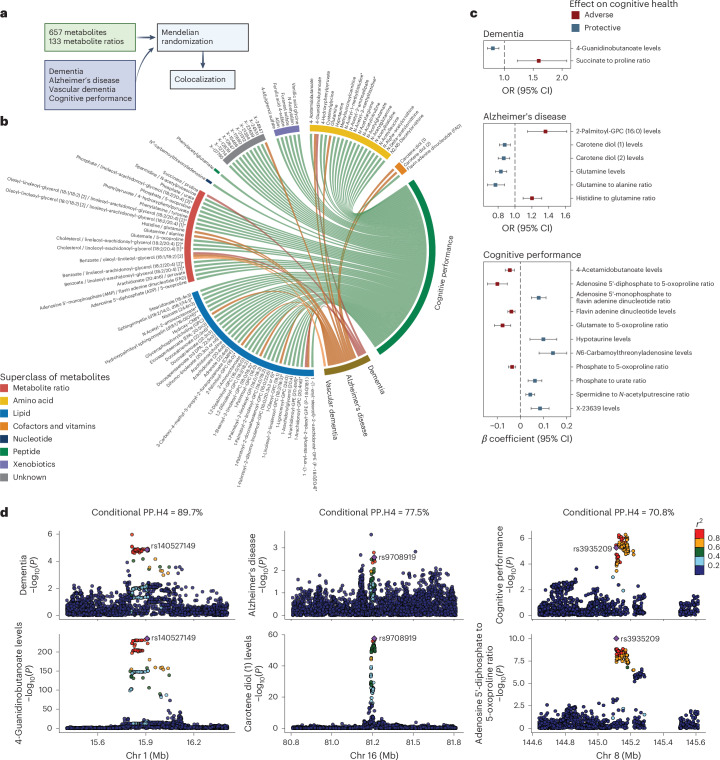
Genetics enables the inference of putative causal relationships between plasma metabolites and cognitive outcomes. **a**, Schematic of the two-sample MR and colocalization analyses. Genetic instruments were selected for 657 metabolites and 133 metabolite ratios from a published GWAS. Summary statistics for overall dementia, AD, vascular dementia and cognitive performance were also obtained from published GWASs (Methods). Two-sample MR was performed to identify putative causal relationships between metabolites or ratios and cognitive outcomes, followed by colocalization analysis for the causal associations with an FDR < 0.05. **b**, Identification of numerous putative causal interrelationships of various metabolites, metabolite ratios and cognitive outcome using genetic instruments. The chord diagram displays causal relationships with an FDR < 0.05 from MR analyses using Wald ratio, inverse variance-weighted or MR Egger methods, where each arc represents an identified link between a metabolite or ratio and a cognitive outcome. Arcs and nodes are color coded by the HMDB superclasses. **c**, Colocalization analyses strengthening evidence of causality, suggesting that the identified putative causal relationships between metabolites or ratios and cognitive outcomes have potential shared causal variants and biology. Putative causal relationships, represented by the odds ratios (ORs) or *β* coefficients with 95% CIs, are shown for associations with FDR < 0.05 and colocalization signals. Bayesian colocalization analysis was performed within the ±500-kb region around a genetic instrument (Methods). If multiple instruments were used, a causal association was reported if the metabolite or ratio and cognitive outcomes colocalized at least one genetic locus. Colocalization signals were reported for a locus if the conditional probability of colocalization, PP.H4/(PP.H3 + PP.H4), was >70%, where PP.H3 is posterior probability that the two traits have independent causal variants and PP.H4 is the posterior probability that the two traits share a single causal variant. **d**, Regional genetic association plots providing evidence of potential shared causal variants affecting both metabolites or ratios and cognitive outcomes at specific genetic loci. The plots display genetic association results for metabolites or ratios and cognitive outcomes at three colocalized loci with PP.H4/(PP.H3 + PP.H4) > 70%. Each plot is annotated with the genetic instrument and dots are color coded according to their linkage disequilibrium with the instrumental variant. The −log_10_(*P*) values for both metabolites or ratios and cognitive outcomes were obtained from the original GWASs. The sample sizes for the original GWASs are as follows: metabolites or ratios (*n* = 8,299), cognitive performance (*n* = 257,841), dementia (5,933 cases and 166,584 controls), AD (90,338 cases and 1,036,225 controls) and vascular dementia (881 cases and 211,508 controls) (Methods). All statistical tests were two sided. Panel **a** created using BioRender.com. Source data

Last, genetics can offer mechanistic insights by using variants as instrumental variables to test whether associations between metabolites and cognitive outcomes are causal using the MR approach. To maximize the statistical power and mitigate biases, we implemented a two-sample MR design leveraging data from published GWASs and selected genetic instruments for 657 metabolites and 133 ratios of metabolite pairs sharing an enzyme or transporter (reflecting metabolic flux; Supplementary Tables 24 and 25)^11^. Cognitive outcomes included overall dementia, AD, vascular dementia and cognitive function (Fig. 5a and Methods).

We identified 99 significant causal relationships involving 95 metabolites or ratios across all 4 outcomes at an FDR < 0.05 (Fig. 5b and Supplementary Table 26). A colocalization analysis was further performed to prioritize metabolite–outcome pairs with potential shared genetic basis and biology; we identified 19 pairs with a conditional posterior probability of colocalization >70% under a single causal variant assumption (Fig. 5c and Supplementary Table 27).

A notable finding was the protective causal effect of 4-guanidinobutanoate (4-GBA) on dementia, supported by colocalization signals (Fig. 5d); 4-GBA is a gamma-aminobutyric acid (GABA)-related metabolite involved in inhibitory neurotransmission^42^ and may counteract excitotoxicity, a known contributor to dementia pathogenesis^43^. The genetic instrument, rs140527149, is mapped to *AGMAT*, which encodes agmatinase, an enzyme involved in the degradation of agmatine in the brain^44^. Evidence suggests that agmatine may have therapeutic potential for AD by modulating Aβ production, aggregation and clearance^45^. Our findings highlight potential shared pathways involving 4-GBA, GABA and agmatine in dementia pathology. Carotene diol (1) and (2), naturally occurring carotenoids with potent antioxidant properties^46^, showed protective effects against AD, consistent with randomized controlled trial evidence causally linking carotenoid intake to reduced cognitive decline^47^. Building on studies showing altered glutamine levels in the brain and cerebrospinal fluid of individuals with AD^48^, our findings also highlighted glutamine’s potentially neuroprotective role in AD (Supplementary Fig. 9). For cognitive function, *N*^6^-carbamoylthreonyladenosine exhibited a strong beneficial effect, with colocalization signals allowing for multiple causal variants (Methods and Supplementary Table 28). A number of causal relationships were identified between metabolites involved in ATP metabolism, suggesting a causal role for the interplay between disrupted energy metabolism and oxidative stress in the pathophysiology of cognitive decline (Supplementary Text). Future investigations into these pathways could inform targeted therapeutic strategies.

## Discussion

Elucidating the pathogenic mechanisms underlying ADRD and identifying modifiable factors capable of targeting these mechanisms are critical for prevention. Given the strong genetic basis of ADRD, accounting for genetic predisposition is essential when investigating these mechanisms. In this study, we leveraged up to 34 years of follow-up and multiomics data from the NHS, with independent replication in the HPFS, to comprehensively examine gene–metabolite interactions in relation to ADRD risk. We identified distinct plasma metabolomic profiles of dementia risk among *APOE4* homozygotes. Beyond *APOE4*, we found that the metabolite–dementia associations are also modified by other common AD/ADRD risk variants implicated in cholesterol homeostasis, mitochondrial function, APP production and extracellular matrix integrity. Notably, adherence to the MedDiet contributed to cognitive health in an *APOE4*-dependent manner, potentially mediated by metabolites. We further demonstrated that incorporating metabolites into predictive models alongside traditional and genetic predictors improved the prediction performance. Our two-sample MR analysis further supported the causal effects of specific metabolites, such as 4-guanidinobutanoate, carotene diol and glutamine, on cognitive outcomes.

ADRD has long been considered a metabolic disease, largely due to the central role of *APOE4* in lipid transport and metabolism^6^. Emerging evidence indicates that the metabolomic contributions to ADRD risk are modulated by *APOE4* status^17–20^. A recent study further highlights that *APOE4* homozygosity represents a distinct genetic subtype which exhibits significantly higher levels of AD pathological markers at a younger age^7^. Our study extends those findings, revealing that *APOE4* not only distorts lipid metabolism but also affects other pathways, including betaine and the urea cycle (for example, citrulline) metabolism. These findings suggest a broader role for *APOE4*, implicating its involvement in neuroinflammation, gut-derived choline metabolism and neurotoxic pathways that elevate ADRD risk^5,49^. Furthermore, our findings, aligned with the recent study^7^, indicate that *APOE4* homozygosity uniquely alters the plasma metabolome and its associations with ADRD risk, underscoring the necessity of distinguishing homozygotes from heterozygotes in investigations of *APOE4*’s role in AD pathology. Although *APOE4* homozygotes exhibit a high penetrance of AD biology, not all individuals progress to clinical AD^6^. This resilience presents an opportunity to identify protective mechanisms specific to *APOE4* homozygosity. Our findings highlight the potential for preventive strategies targeting specific metabolic pathways in this high-risk group. Beyond *APOE4*, examination of other common risk variants identified additional findings pointing to broader pathogenic mechanisms.

Consistent with the results from the PREDIMED trial^25^, we found that long-term adherence to the MedDiet was strongly associated with lower ADRD risk. We further examined whether this association varied by genetic predisposition. A key distinction of our study, compared to previous studies with mixed findings^50–52^, is that we went beyond the binary classification of *APOE4* carrier status and found particularly strong associations only among *APOE4* homozygotes. The strongest association in *APOE4* homozygotes may reflect more effective modulation of ADRD-related metabolic profiles by the MedDiet in this genotype. Thus, our study not only identifies mechanistic insights, but also proposes actionable prevention strategies targeting these pathways. This has important implications for public health messaging, highlighting the overall benefit of adhering to the MedDiet for ADRD prevention, as well as the potential for targeted interventions in genetically vulnerable populations. In addition, our findings underscore the value of metabolites in predicting ADRD risk decades before disease onset. Last, our two-sample MR analysis corroborated established mechanisms in ADRD pathology, such as the role of GABAergic signaling, reinforcing causality derived from mechanistic studies and randomized controlled trials, including evidence of carotenoids’ neuroprotective effects. We also identified new causal biomarkers, including metabolites involved in glutamine, urate and hypotaurine metabolism (Supplementary Text). Collectively, these findings suggest potential druggable targets and inform pathways for more effective and precise prevention and treatment strategies for ADRD.

Our study has limitations. It was conducted in well-educated individuals of European ancestry, which may limit generalizability but enhances internal validity. Ascertainment bias is possible given the exclusion of participants with dementia at baseline, although the baseline age was relatively young and *APOE4* homozygotes did not differ significantly in health status, making major bias unlikely. Dementia outcomes were based on self-reported physician diagnoses and death records, which may introduce misclassification, but validity is supported by participants’ professional backgrounds, strong associations with known genetic factors and p-tau217 and consistency across data sources. Although omics-based models are not yet widely implemented clinically, emerging tools such as PRSs and aging clocks show translational promise (Supplementary Text). Despite these limitations, our large, prospective design enabled robust examination of gene–metabolite–diet interactions over decades of follow-up. Importantly, key findings in women were independently validated in men, supporting generalizability across sexes, particularly relevant given the sex-based differences in both metabolite profiles^53^ and dementia risk^54^.

In conclusion, our study highlights the substantial influence of genetic variants, particularly *APOE4* homozygosity, on plasma metabolites and their associations with ADRD risk. These genetic effects are widespread across the plasma metabolome and our findings identify the MedDiet as a promising approach to mitigate genetically dependent ADRD risk by targeting a broad spectrum of metabolic pathways. Moreover, this work provides a uniquely valuable resource for advancing the early prediction of ADRD risk through emerging omics-based biomarkers and identifying causal mechanisms underlying ADRD risk, which hold potential as prevention targets and druggable pathways.

## Methods

### Ethics statement

This study included de-identified data from participants who had consented to the use of their anonymized information for research purposes. Participants were not financially compensated for their participation. Approvals for the study protocol of the NHS and the HPFS were granted by the institutional review boards of Brigham and Women’s Hospital and the Harvard T.H. Chan School of Public Health (institutional review board protocol nos. 1999P011114/BWH for the NHS and HSPH 22067-102 for the HPFS).

### Study populations

The NHS is a prospective cohort study that enrolled 121,700 US female registered nurses aged 30–55 years in 1976^55^. The participants were followed biennially to collect information on diet, lifestyle, medication use and newly diagnosed diseases through mailed self-administered questionnaires. We included 4,215 women who were aged <75 years and free from dementia, Parkinson’s disease, stroke and cancer at baseline when the blood samples were collected (1989–1992); the blood was assayed for genetics and metabolomics, as described in detail below.

The HPFS is a prospective cohort study that enrolled 51,529 US male health professionals aged 40–75 years in 1986. Similar to the NHS, the participants were followed biennially via mailed questionnaires. We included 1,490 men who met the same inclusion and exclusion criteria at baseline (1993–1996), when the blood samples were collected, as a replication cohort.

### Ascertainment of dementia and assessment of objective cognitive function

The participants were followed from the baseline to 2023 for a composite dementia endpoint, which included self-reported dementia and deaths due to dementia. Participants self-reported a physician diagnosis of AD or other forms of dementia (ADRD) every 2 years via questionnaires. Deaths were identified through state vital statistics records, the National Death Index, family reports and the postal system. A study physician reviewed medical records and death certificates to determine whether dementia was listed as the primary or contributing cause of death. Our system for death ascertainment and death cause adjudication is validated and >98% of deaths were identified^56^.

Objective cognitive function was assessed through a telephone cognitive interview in a subset of 1,037 NHS participants aged ≥70 years, with four telephone interviews conducted between 1995 and 2008. The telephone cognitive battery initially included the TICS, which is a telephone adaptation of the Mini-Mental State Examination^57^. Five other tests were added later, including (1) immediate recall of the East Boston Memory Test (EBMT), (2) delayed recall of the EBMT, (3) delayed recall of the TICS ten-word list, (4) a test of verbal fluency and (5) the digit span backward test. We assessed three cognitive measures, including the TICS score and the composite scores for global cognition and verbal memory. Global cognition included all six tests. Verbal memory included four tests: immediate recall of the ten-word list in the TICS, delayed recall of the TICS ten-word list and immediate and delayed recalls of the EBMT. To calculate the composite scores for global cognition and verbal memory, we first calculated *z*-scores for each test at each measurement, based on the baseline mean and the s.d. We then averaged these *z*-scores across all relevant tests at each measurement and calculated the overall mean value across all measurements.

### Genotyping, *APOE4* genotype, other AD/ADRD variants and the PRS for ADRD

Details of genotyping, QC and imputation for the NHS and HPFS are described elsewhere^58^ (https://github.com/cturman15/ChanGWASlab) and in Supplementary Text. Blood samples were genotyped using one of six genotyping platforms. We restricted the samples to inferred European ancestry based on genetic PCs. Post-QC data were imputed using the 1000 Genomes Phase 3 v5 reference panel. *APOE4* genotype was determined using two SNPs: rs429358 and rs7412. *APOE4* homozygotes carried C/C alleles for both SNPs and *APOE4* heterozygotes carried C/T for rs429358 and C/C or C/T for rs7412. All other combinations of alleles were considered *APOE4* noncarriers. The average imputation quality score for rs429358 and rs7412 was 0.91 and 0.87 across genotyping platforms, respectively. We calculated PRSs for ADRD using weights from two published studies^9,21,59^. The PRS developed by Bellenguez et al. (PGS002280) comprised 83 variants and excluded any variants from the *APOE* region^9^. The PRS developed by Zhang et al. (PGS000334) comprised 22 variants and included the two *APOE* variants^21^. Only variants available in the imputed genetic data were included in the PRS calculation (Supplementary Table 11). The PRS was calculated as a weighted sum of the effect allele dosage across all included variants for each individual and was standardized to have a mean of 0 and an s.d. of 1 within each genotyping platform to maximize comparability. We further extracted individual variants from these two PRSs, which were identified in previous GWASs of AD/ADRD. Duplicated variants and those with a minor allele frequency <0.01 were removed from the combined list, followed by linkage disequilibrium pruning (*r*^2^ < 0.1 with the 1000 Genomes European population as the linkage disequilibrium reference) using the SNPclip function in the LDlinkR package in R, leaving a total of 73 variants for subsequent analyses (Supplementary Table 12).

### Metabolomic profiling

Plasma metabolomic profiling was performed for nested case–control studies within the NHS and HPFS using high-throughput LC–MS techniques at the Broad Institute of MIT and Harvard (Cambridge, MA, USA). Additional details of metabolomic profiling are provided in Supplementary Text. For metabolites with <25% missing data in an individual study, missing values were imputed with half of the minimum measured value for that metabolite in that study; metabolites with <100 samples were removed. A probit transformation was applied to metabolites within each study to correct for batch effects, reduce the impact of skewed distributions and heavy tails on the results and scale the metabolite values to the same range. After merging the metabolite data with other types of data (*n* = 4,215 for the NHS and *n* = 1,490 for the HPFS), we excluded metabolites with ≥90% missing values among dementia cases with genetic data. We further excluded metabolites with an intraclass correlation coefficient <0.4 or coefficient of variation in the top 10 percentiles of all remaining metabolites. A total of 401 metabolites was included in the final analysis for the NHS, of which 254 were available in the HPFS. We created another set of metabolomics data for the NHS by selecting those with ≥100 samples and <25% missing values from the 401 metabolites in the final dataset, followed by RF imputation with 100 trees using the missRanger package in R; 237 metabolites were retained for subsequent analyses of the overall metabolomic profile that required no missing data. Using the same approach, we retained 164 non-missing metabolites in the HPFS.

### Dietary assessment and the MedDiet score

Dietary intakes were assessed using SFFQs. The validity and reproducibility of the SFFQs have been demonstrated in previous publications^60^. We calculated the average dietary intakes from the first dietary assessment (1980 for the NHS and 1986 for the HPFS) to the SFFQ closest to the blood draw time to reflect the long-term diet. The MedDiet was assessed using the Alternate Mediterranean Diet Score, which was calculated based on nine components^61^. For vegetables, fruit, nuts, whole grains, legumes, fish and the ratio of monounsaturated to saturated fat, a score of 1 was given if the intake was at or above the SFFQ-specific median; otherwise, 0 was given. For red and processed meat consumption, a score of 1 was assigned if the intake was below the SFFQ-specific median; otherwise, a score of 0 was assigned. For alcohol intake, a score of 1 was assigned if the intake was between 5 and 15 g d^−1^; otherwise, a score of 0 was assigned. The scores of individual components were summed to obtain the overall MedDiet index, which ranged from 0 to 9.

### Assessment of global impacts of genetics and MedDiet on plasma metabolome

We assessed the overall correlation of genetic data, the MedDiet index and the plasma metabolome in the NHS. Pearson’s correlation coefficients were calculated for each of the 401 metabolites with genetic PC1 and PC2. Correlation coefficients were then ranked from highest to lowest for PC1 and PC2, respectively. The overall rank was determined by summing the ranks of PC1 and PC2 and ranking them from lowest to highest.

Leveraging the 237 metabolites with no missing values in the NHS (see above), we assessed the overall correlation between metabolites and the MedDiet index, as well as the predictive performance of metabolites on the MedDiet index. Pearson’s correlation coefficients were calculated for the MedDiet index and its individual components (monounsaturated and saturated fat calculated separately) with metabolites PC1 and PC2 calculated from the 237 metabolites. RF regression was performed to evaluate the predictive performance of the metabolites on the MedDiet index. The dataset was first randomly split into training (60%) and test (40%) sets. A dichotomized MedDiet index outcome was derived from the top and bottom quartiles of the continuous score. RF regression with five-fold cross-validation was performed on the training set to tune the parameter using the train and trainControl functions in the caret package in R. The tuned model was then applied to the test set to evaluate performance using the AUC. The same approach was applied to the HPFS to assess the predictive performance of metabolites on the MedDiet index.

### Interaction analyses of plasma metabolome and genetic variation in relation to dementia risk and cognitive function

Cox PH models were fitted to assess the associations between each of the 401 metabolites and the time-to-event outcome of dementia among 4,215 women in the NHS using the coxph function in the survival package in R. Details of covariate assessment and adjustment are provided in Supplementary Text. The FDR correction was applied to the *P* value for metabolites using the Benjamini–Hochberg approach, with an FDR < 0.05 considered statistically significant. The same approach and FDR threshold were used for all other analyses involving multiple testing corrections in this study, including all interaction tests. All statistical tests in this study were two sided. For the interaction analysis of metabolites and *APOE4* status, interaction terms between *APOE4* carrier status (or *APOE4* heterozygote and homozygote) and the metabolite were added to the model, along with ADRD PRS (excluding the *APOE* region) and its interaction term with the metabolite. For the models of *APOE4* heterozygote and homozygote, FDR correction was applied jointly to the interaction *P* value of the two *APOE4* terms. In addition, likelihood ratio tests were performed comparing the model with and without the *APOE4* interaction terms. For the interaction analysis of metabolites with ADRD PRS or other AD/ADRD variants, the relevant gene–metabolite interaction terms (PRS or effect allele dosage) were added to the model, along with *APOE4* heterozygote and homozygote. All interaction models were additionally adjusted for the top four genetic PCs and genotyping platforms. For the subgroup analyses within *APOE4* noncarriers, carriers and heterozygotes, the models were further adjusted for the ADRD PRS (excluding the *APOE* region), the top four genetic PCs and genotyping platforms. For *APOE4* homozygotes, the models were adjusted for only continuous covariates, ADRD PRS and the top four genetic PCs due to the limited sample size. For analyses within ADRD PRS tertiles (defined using genetic PC-adjusted scores), no additional covariate was adjusted.

Generalized linear models (Gaussian family) were fitted to assess the association between each objective cognitive function score and each of the 401 metabolites among a subset of 1,037 women with cognitive function measurements using the glm function of the stats package in R. The models were adjusted for the same covariates as in the dementia risk analysis. Interaction analysis was performed for *APOE4* carrier status with the same additional covariates as in the dementia risk analysis. *APOE4* carriers were not further stratified into heterozygotes and homozygotes due to data sparsity among homozygotes in this subset. FDR correction was applied to the interaction *P* value per cognitive outcome. Interaction analysis with other genetic factors or subgroup analysis was not performed due to the limited power.

As a replication, interaction analysis of the 254 available metabolites was conducted in 1,490 men from the HPFS, with additional adjustment for profession. Due to data sparsity in non-missing values for each metabolite, only interactions with *APOE4* carrier status were assessed.

### Associations of MedDiet adherence with dementia risk and cognitive function

In the analysis of the associations of the MedDiet index score with dementia risk and objective cognitive function, we leveraged data from the full NHS cohort and excluded participants who had dementia, Parkinson’s disease, stroke, cancer or missing components for the MedDiet index at baseline, which was 1980 for the dementia endpoint and 1994 for the objective cognitive function. For the analysis of the objective cognitive function, we further excluded participants aged <70 years at baseline. A total of 86,740 participants were included in the analysis of dementia risk and 16,244 participants in the analysis of objective cognitive function.

Cox PH model was fitted to prospectively assess the association between the continuous MedDiet index and the time-to-event outcome of dementia risk. Details of covariate adjustment are provided in Supplementary Text. A cubic spline regression model was fitted to assess the nonlinear trend. Subgroup analyses were performed by *APOE4* genotype and tertiles of ADRD PRSs.

Generalized linear models were fitted to assess the association between the continuous MedDiet index and the objective cognitive function scores. These models adjusted for the same covariates as the dementia risk model. Subgroup analyses were performed by *APOE4* genotype and tertiles of ADRD PRSs.

As a replication, a total of 43,500 male participants from the HPFS were included in the analysis of the association between MedDiet adherence and dementia risk, applying the same exclusion criteria as in the NHS. The same Cox PH and cubic spline models were fitted, with additional adjustment for profession. Subgroup analyses were performed by *APOE4* genotype.

### Associations between MedDiet adherence and plasma metabolome by genetic subgroups

Generalized linear models were fitted to assess the association between the MedDiet index and each of the 401 metabolites among 4,215 women in the NHS, adjusting for the same covariates as the above linear model for cognitive function and metabolites. Subgroup analyses were performed by *APOE4* genotype and tertiles of ADRD PRS (excluding *APOE* region). As a replication, the same models were fitted to assess the association between the MedDiet index and each of the 254 overlapping metabolites among 1,490 men in the HPFS, additionally adjusted for profession.

### Mediation effect of metabolites on the association between MedDiet adherence and dementia risk by *APOE4* carrier status

We conducted a mediation analysis to quantify the extent to which metabolites mediate the association between MedDiet adherence and dementia risk in the NHS. Metabolites associated with both dementia risk (*P* < 0.05) and MedDiet adherence (FDR < 0.05) were selected as candidate mediators, followed by orthogonal filtering using a correlation threshold of *r* < 0.5 to exclude highly correlated metabolites, resulting in seven metabolites: allantoin, C16:1 CE, C18:0 SM, 1-methylguanine, 1,7-dimethyluric acid, C34:5 phosphatidylcholine plasmalogen and piperine. Regression-based mediation analyses were then conducted by comparing a full model (including both MedDiet and selected metabolites) with a reduced model (excluding metabolites), with dementia risk as the dependent variable to quantify the proportion of the effect of MedDiet adherence on dementia risk, explained by these selected metabolites in the full dataset, as well as stratified by *APOE4* carrier status.

### Prediction of dementia risk and cognitive function

Cox PH models were built for predicting dementia risk among 4,215 women in the NHS. The dataset was first randomly split into training (60%) and test (40%) sets, including within *APOE4* subgroups. A 15-year outcome-free attainment was defined by censoring participants without event by year 15. We built four prediction models: the baseline model included age, family history of dementia, educational attainments of nurses, smoking status, history of depression or regular antidepressant drug use and the MedDiet index; the *APOE4* model further included the *APOE4* heterozygote and homozygote indicators, the PRS model the ADRD PRS (excluding the *APOE* region) and the metabolite model the selected metabolites, including 12 metabolites for predicting overall outcomes and 4 metabolites for predicting the 15-year outcome-free attainment (Supplementary Text and Supplementary Table 22). *APOE4* predictors were not included in the models for *APOE4* subgroups. All models were built in the training set and evaluated on the test set. Time-dependent AUC was calculated based on an incident/dynamic version of sensitivity and specificity within distinct risk sets, defined as groups of participants who remained at risk for developing dementia at specified time points, which provides a dynamic view of model performance across the follow-up period^62^, using the risksetAUC function in the risksetROC package in R. Harrell’s C-index^63^ was calculated to quantify the overall discriminative ability of the model by assessing how well the model can rank individuals by their risk of the event using the survival package in R. Feature contributions were quantified by the SHAP value^64^ for Cox PH model in predicting overall and 15-year dementia risk, including the full list of predictors using the fastshap package in R. SHAP values were calculated for each category of predictors by summing the SHAP value of all predictors in that category.

RF models with 500 trees were built for predicting the dichotomized outcomes of the highest versus the lowest tertile of the continuous cognitive function scores, using the randomForest package in R. The same predictors and analysis strategy were used as in the prediction models for dementia risk. The AUC was calculated to evaluate the model performance in the test set.

As a replication, Cox PH models were built to predict dementia risk among 1,490 men in the HPFS. The same modeling approaches were applied, with profession included in the baseline model instead of education level. Due to data sparsity, prediction models were constructed only for the overall dementia risk in the full dataset. The 164 RF-imputed metabolites with no missing values were considered as candidate predictors and 5 metabolites were selected in the final model, following the same procedure used in the NHS (Supplementary Table 22).

### Two-sample MR analysis

#### GWAS sources

Two-sample MR analysis^65^ was conducted to assess the causal relationships between plasma metabolites and cognitive outcomes using published GWAS summary statistics. GWAS summary statistics for the exposures, including plasma metabolites and metabolite ratios, were obtained from ref. ^11^ and for the cognitive outcomes were obtained from GWASs of overall dementia^66^, AD^8^, vascular dementia^66^ and cognitive performance^67^. There was no overlap of the participants between the GWAS cohorts of the exposures and the outcomes. All GWAS populations were of European ancestry.

#### Genetic instruments

In the original study^11^, GWASs were performed for 1,091 plasma metabolites and 309 metabolite ratios. In this study, we included all the variant–metabolite and variant–metabolite ratio pairs selected by the original study for MR analyses, with additional selection applied to metabolites and ratios not included in the original set (Supplementary Text). A total of 1,431 variant–metabolite pairs for 657 metabolites and 186 variant–metabolite pairs for 133 metabolites ratios were selected for the MR analysis.

#### MR analysis

We used three MR methods to assess the causal effect of each metabolite or ratio on each cognitive outcome. Wald ratio method was used when there was only one genetic instrument. The inverse variance-weighted method was used when there were at least two instruments. The MR Egger method was used when there were at least three instruments and potential pleiotropy was detected. MR analysis was performed using the mr function in the TwoSampleMR package in R. Details of the sensitivity analyses are described in Supplementary Text.

### Colocalization analysis

Colocalization analysis was performed for exposure–outcome pairs that passed FDR < 0.05 in the MR analysis. For each pair, Bayesian colocalization analysis was performed in the ±500-kb region around each genetic instrument, restricting to variants with minor allele frequency <0.01, using the coloc.abf function with default prior probabilities in the coloc package in R. Colocalization signals were reported for a locus if the conditional probability of colocalization, PP.H4/(PP.H3 + PP.H4), was >70%, where PP.H3 is posterior probability that the two traits have independent causal variants and PP.H4 is the posterior probability that the two traits share a single causal variant. These colocalized exposure–outcome pairs were considered to have putative causal relationships. If there were instruments at multiple loci for a pair, the causal relationship was reported if the exposure and outcome were colocalized at at least one genetic locus. The coloc.abf function assumes that there is a single causal variant for each trait. We further conducted a sensitivity analysis that relaxed the single causal variant assumption using the coloc.susie function in the coloc package in R, which calculates posterior probabilities under the assumption of multiple causal variants.

### Reporting summary

Further information on research design is available in the Nature Portfolio Reporting Summary linked to this article.

## Online content

Any methods, additional references, Nature Portfolio reporting summaries, source data, extended data, supplementary information, acknowledgements, peer review information; details of author contributions and competing interests; and statements of data and code availability are available at 10.1038/s41591-025-03891-5.

## Supplementary information

Supplementary Information Supplementary Text and Figs. 1–9.

Reporting Summary

Supplementary Tables Supplementary Tables 1–28.

Supplementary Data 1–9 Statistical source data for Supplementary Figs. 1–9.

## Source data

Source Data Fig. 1 Statistical source data.

Source Data Fig. 2 Statistical source data.

Source Data Fig. 3 Statistical source data.

Source Data Fig. 4 Statistical source data.

Source Data Fig. 5 Statistical source data.

Source Data Extended Data Fig. 1 Statistical source data.

Source Data Extended Data Fig. 2 Statistical source data.

Source Data Extended Data Fig. 3 Statistical source data.

Source Data Extended Data Fig. 4 Statistical source data.

Source Data Extended Data Fig. 5 Statistical source data.

Source Data Extended Data Fig. 6 Statistical source data.

Source Data Extended Data Fig. 7 Statistical source data.

Source Data Extended Data Fig. 8 Statistical source data.

Source Data Extended Data Fig. 10 Statistical source data.

## Data Availability

As informed consent was gained from the participants, all the individual-level data from the NHS and the HPFS are available through a request for external collaboration and upon approval of a letter of intent and a research proposal. Details on how to request external collaborations with the NHS can be found at https://nurseshealthstudy.org/researchers (contact principal investigator: A.H.E., email: nhahe@channing.harvard.edu) and with the HPFS at https://sites.sph.harvard.edu/hpfs/for-collaborators (contact principal investigator: L. Mucci, email: lmucci@hsph.harvard.edu). Source data are provided with this paper.

## Code availability

Analysis-specific programs are publicly available via GitHub at https://github.com/DW-Group/Gene_Metabolites_MedDiet_Dementia.
