# Author Correction: Interplay of genetic predisposition, plasma metabolome and Mediterranean diet in dementia risk and cognitive function

**DOI:** 10.1038/s41591-025-04025-7

**Published:** 2025-10-02

**Authors:** Yuxi Liu, Xiao Gu, Yanping Li, Fenglei Wang, Chirag M. Vyas, Cheng Peng, Danyue Dong, Yuhan Li, Yu Zhang, Yin Zhang, Oana A. Zeleznik, Jae H. Kang, Molin Wang, Frank B. Hu, Walter C. Willett, Olivia I. Okereke, A. Heather Eliassen, Peter Kraft, Meir J. Stampfer, Dong D. Wang

**Affiliations:** 1 Channing Division of Network Medicine, Department of Medicine, Brigham and Women’s Hospital and Harvard Medical School, Boston, MA USA; 2 Department of Epidemiology, Harvard T.H. Chan School of Public Health, Boston, MA USA; 3 Broad Institute of MIT and Harvard, Cambridge, MA USA; 4 Department of Nutrition, Harvard T.H. Chan School of Public Health, Boston, MA USA; 5 Department of Psychiatry, Massachusetts General Hospital and Harvard Medical School, Boston, MA USA; 6 Department of Biostatistics, Harvard T.H. Chan School of Public Health, Boston, MA USA; 7 Division of Cancer Epidemiology and Genetics, National Cancer Institute, Rockville, MD USA

**Keywords:** Alzheimer's disease, Genetic association study, Epidemiology, Predictive markers, Risk factors

Correction to: *Nature Medicine* 10.1038/s41591-025-03891-5, published online 25 August 2025.

In the version of this article initially published, the copyright was listed under “The Author(s)” and has now been amended to “This is a U.S. Government work and not under copyright protection in the US; foreign copyright protection may apply” in the HTML and PDF versions of the article.

