# Supplementary material for: Interplay of genetic predisposition, plasma metabolome and Mediterranean diet in dementia risk and cognitive function
Source: Nat Med. 2025 Aug 25;31(11):3790–800. doi: 10.1038/s41591-025-03891-5 (PMC12618253; doi:10.1038/s41591-025-03891-5)
Supplement: Supplementary file 1 — Supplementary Text and Figs. 1–9. [file 41591_2025_3891_MOESM1_ESM.pdf]

nature medicine

Supplementary information https://doi.org/10.1038/s41591-025-03891-5

Interplay of genetic predisposition, plasma metabolome and Mediterranean diet in dementia risk and cognitive function

In the format provided by the authors and unedited

Supplementary Text

Supplementary Methods

Genotyping, quality control, and imputation

Blood samples were genotyped as part of nested case-control studies or sub-cohorts within the Nurses' Health Study (NHS) and Health Professionals Follow-Up Study (HPFS), originally designed for genome-wide association studies (GWAS), using one of the six genotyping platforms: Affymetrix 6.0, Illumina HumanHap, Illumina OmniExpress, HumanCore Exome, Illumina OncoArray, and Infinium Global Screening Array. Genotyping data were harmonized and merged by platform. We excluded variants with call rate <95% or Hardy-Weinberg equilibrium $p < 1\times10^{-6}$ and samples with sex discordance, extreme heterozygosity, or call rate <90%. We further restricted the samples to inferred European ancestry based on genetic principal components (PCs). Post-quality control (QC) genotype data were imputed using the 1000 Genomes Phase 3 version 5 (1000G) reference panel. We further excluded variants with an imputation quality score <0.3. The final genetic PCs were calculated using ~38,000 post-imputation overlapping variants across the six platforms. Samples were projected onto the PCs of the 1000G European population, ensuring that PCs are comparable across all participants and platforms.

Metabolomic profiling

Plasma metabolomic profiling was performed for nested case-control studies within the NHS and HPFS using high-throughput liquid chromatography-mass spectrometry (LC-MS/MS) techniques at the Broad Institute of MIT and Harvard (Cambridge, MA, USA). Three LC-MS/MS methods that were designed to measure polar metabolites and lipids, and free fatty acids were used, as detailed in previous studies.[1,2] For each method, pooled plasma reference samples were included every 20 samples, and the results were standardized by calculating the ratio of the sample value to the nearest pooled reference value, then multiplying it by the median of all reference values for the metabolite. In addition, QC samples, blinded to the laboratory, were randomly distributed among the participants' samples and profiled.

Hydrophilic interaction liquid chromatography (HILIC) analyses of water-soluble metabolites in the positive ionization mode were conducted using an LC-MS system comprised of a Shimadzu Nexera X2 U-HPLC (Shimadzu Corp.; Marlborough, MA) coupled to a Q Exactive mass spectrometer (Thermo Fisher Scientific; Waltham, MA). Metabolites were extracted from plasma (10 µL) using 90 µL of acetonitrile/methanol/formic acid (74.9:24.9:0.2 v/v/v) containing stable isotope-labeled internal standards (valine-d8, Sigma-Aldrich; St. Louis, MO; and phenylalanine-d8, Cambridge Isotope Laboratories; Andover, MA). The samples were centrifuged (10 min, 9,000 x g, 4°C), and the supernatants were injected directly onto a 150 x 2 mm, 3 µm Atlantis HILIC column (Waters; Milford, MA). The column was eluted isocratically at a flow rate of 250 µL/min with 5% mobile phase A (10 mM ammonium formate and 0.1% formic acid in water) for 0.5 minute, followed by a linear gradient to 40% mobile phase B (acetonitrile with 0.1% formic acid) over 10 minutes. MS analyses were conducted using electrospray ionization in the positive ion mode using full scan analysis over 70-800 m/z at 70,000 resolution and 3 Hz data acquisition rate. Other MS settings were: sheath gas 40, sweep gas 2, spray voltage 3.5 kV, capillary temperature 350°C, S-lens RF 40, heater temperature 300°C, microscans 1, automatic gain control target 1e6, and maximum ion time 250 ms.

Plasma lipids were profiled using a Shimadzu Nexera X2 U-HPLC (Shimadzu Corp.; Marlborough, MA). Lipids were extracted from plasma (10 µL) using 190 µL of isopropanol

containing 1,2-didodecanoyl-sn-glycero-3-phosphocholine (Avanti Polar Lipids; Alabaster, AL). After centrifugation, supernatants were injected directly onto a 100 x 2.1 mm, 1.7 μm ACQUITY BEH C8 column (Waters; Milford, MA). The column was eluted isocratically with 80% mobile phase A (95:5:0.1 vol/vol/vol 10mM ammonium acetate/methanol/formic acid) for 1 minute followed by a linear gradient to 80% mobile-phase B (99.9:0.1 vol/vol methanol/formic acid) over 2 minutes, a linear gradient to 100% mobile phase B over 7 minutes, then 3 minutes at 100% mobile-phase B. MS analyses were conducted using electrospray ionization in the positive ion mode using full scan analysis over 200-1100 m/z at 70,000 resolution and 3 Hz data acquisition rate. Other MS settings were: sheath gas 50, in source CID 5 eV, sweep gas 5, spray voltage 3 kV, capillary temperature 300°C, S-lens RF 60, heater temperature 300°C, microscans 1, automatic gain control target 1e6, and maximum ion time 100 ms.

Metabolites of intermediate polarity, including free fatty acids and bile acids, were profiled using a Nexera X2 U-HPLC (Shimadzu Corp.; Marlborough, MA) coupled to a Q Exactive (Thermo Fisher Scientific; Waltham, MA). Plasma samples (30 μL) were extracted using 90 μL of methanol containing PGE2-d4 as an internal standard (Cayman Chemical Co.; Ann Arbor, MI) and centrifuged (10 min, 9,000 x g, 4°C). The supernatants (10 μL) were injected onto a 150 x 2.1 mm ACQUITY BEH C18 column (Waters; Milford, MA). The column was eluted isocratically at a flow rate of 450 μL/min with 20% mobile phase A (0.01% formic acid in water) for 3 minutes followed by a linear gradient to 100% mobile phase B (0.01% acetic acid in acetonitril) over 12 minutes. MS analyses were conducted using electrospray ionization in the negative ion mode using full scan analysis over m/z 70-850. Additional MS settings are: ion spray voltage, -3.5 kV; capillary temperature, 320°C; probe heater temperature, 300°C; sheath gas, 45; auxiliary gas, 10; and S-lens RF level, 60. Raw data from orbitrap mass spectrometers were processed using TraceFinder 3.3 software (Thermo Fisher Scientific; Waltham, MA) and Progenesis QI (Nonlinear Dynamics; Newcastle upon Tyne, UK) and targeted data from the QTRAP 5500 system were processed using MultiQuant (version 2.1, SCIEX; Framingham, MA).

Assessments of covariates

Information on educational attainments of nurses and their husbands (NHS only), professions (HPFS only), family history of dementia, menopausal status with hormone use status (ever postmenopausal hormone user vs. others; NHS only), neighborhood socioeconomic status index (nSES, z-standardized in the full NHS or HPFS data[3]), marital status, smoking status (current smoker and past smoker vs. never smoker), systolic blood pressure, history of depression or antidepressant drug use, history of hypertension, and history of hypercholesterolemia were collected from the self-administered questionnaires preceding blood draw. Age, fasting status, and blood draw date were collected from the questionnaires completed at blood draw. Body mass index (BMI) was calculated using the height reported in 1976 and body weight reported closest to blood draw time. Leisure-time physical activity was measured using extensively validated questionnaires.[4] We calculated total energy and alcohol intakes based on semiquantitative food frequency questionnaires.

Cox proportional hazards (PH) models were used to assess associations between each of the 401 metabolites and the time-to-event outcome of dementia among 4,215 females in NHS. Models were adjusted for age, date of blood draw, fasting status, BMI, educational attainments of nurses and their husbands, family history of dementia, menopausal status with hormone use status, physical activity, nSES, marital status, smoking status, systolic blood pressure, history of depression or antidepressant drug use, history of hypertension, history of hypercholesterolemia, total energy intake, the Mediterranean diet (MedDiet) index, and alcohol intake, and stratified by endpoint and case-control status of the nested case-control studies for metabolomics assays. For

the analyses of objective cognitive function, the models additionally adjusted for endpoint and case-control status of the nested case-control studies for metabolomics assays, rather than stratifying by them.

The Cox model assessing the association between the continuous MedDiet index and the time-to-event outcome of dementia risk was stratified by age in months and calendar time in two-year groups and was adjusted for baseline BMI, educational attainments of nurses and their husbands, family history of dementia, menopausal status with hormone use status, physical activity, nSES, marital status, living arrangement, smoking status, history of diabetes, history of depression or antidepressant drug use, and history of hypertension.

Selection of metabolite predictors

The 237 random forest-imputed metabolites with no missing value were considered candidate metabolite predictors. We ran Cox models regressing the time-to-event dementia outcomes, including the overall survival and the 15-year survival, on each of the 237 metabolites, adjusting for the same covariates as in the gene-dependent dementia risk analysis, in the training set (60% from a random split of the full dataset). Metabolites with P <0.05 were selected as predictors.

Selection of genetic instruments for two-sample Mendelian randomization analysis

In the original study by Chen et al.[5], the authors selected genetic instruments for 99 metabolites and 43 ratios passing the following selection criteria: 1) included independent genome-wide significant variants that were assigned to effector genes, 2) removed all variants at the *FADS* locus due to high pleiotropy, and 3) removed all variants in the extended major histocompatibility complex region on chromosome 6 (±500 kb) due to high pleiotropy. In the present study, we included all the variant-metabolite and variant-metabolite ratio pairs selected by the original study. Additionally, for those metabolites and ratios with no instrumental variants selected by the original study, we used less stringent selection criteria to include all independent genome-wide significant variants (n =1,702 for metabolites and n =247 for metabolite ratios) identified in the original publication using GCTA-COJO[6]. After excluding variants without rsIDs, 1,591 candidate genetic instruments for additional 575 metabolites and 230 candidate genetic instruments for additional 94 metabolite ratios were selected with the summary statistics obtained from the supplementary tables in Chen et al. We did not restrict to those with effector genes as genetic variants that do not directly modulate the transcription or splicing of protein-coding genes may still be valid instruments for Mendelian randomization (MR) purpose; restricting to only those mapped to effector genes may miss potential signals that either linked to biology of noncoding genes or unknown biology. Horizontal pleiotropy was also systematically assessed in later steps. Next, we extracted the candidate instruments from the outcome data using the extract_outcome_data function in the TwoSampleMR package in R for outcomes that were available in the MRC IEU OpenGWAS project database (https://gwas.mrcieu.ac.uk/), including overall dementia, vascular dementia, and cognitive performance (accession date: 5/12/2024).[7-9] If a variant was not present in the outcome data, a linkage disequilibrium (LD) proxy for the variant with r^2 >0.8 was searched for in the outcome data using the 1000G European population as the LD reference. If no LD proxy was found, the variant was removed from the instrument list. For the Alzheimer's disease GWAS (Wightman et al.[10]) for which the summary statistics were not available in the MRC IEU OpenGWAS project database when we accessed it, we obtained the GWAS meta-analysis summary statistics excluding UK Biobank and 23andMe from https://ctg.cncr.nl/software/summary_statistics; candidate genetic instruments with matching rsIDs in the outcome summary statistics were extracted from the outcome data. We then

harmonized the selected variants in the exposure and outcome data to ensure that the effect of a variant on the exposure and the outcome corresponded to the same allele; the forward strand alleles were inferred using the allele frequency. Harmonization was performed using the harmonise_data function in the TwoSampleMR package in R. A total of 1,431 variant-metabolite pairs for 657 metabolites and 186 variant-metabolite ratio pairs for 133 metabolites ratios were finally selected.

Sensitivity analysis for the two-sample MR analysis

Horizontal pleiotropy was evaluated for exposure-outcome pairs with at least 3 instruments using the mr_pleiotropy_test function in the TwoSampleMR package in R. If potential pleiotropy was detected (MR Egger P <0.05), the estimates from MR Egger were used for the causal relationship for the exposure-outcome pair; otherwise the estimates from the inverse variance weighted (IVW) method were used. Steiger directionality test was performed using the directionality_test function in the TwoSampleMR package in R to evaluate whether the causal direction was from the exposure to the outcome by testing whether the variance explained in the exposure by the instrumental variant is greater than the outcome. We excluded those exposure-outcome pairs with incorrect directionality, i.e., causal direction from outcome to exposure (Steiger P <0.05). Heterogeneity test was performed using the mr_heterogeneity function in the TwoSampleMR package in R to evaluate the heterogeneity of the MR estimates across instruments for IVW and MR Egger. We did not exclude any exposure-outcome pairs based on the heterogeneity test results as the presence of heterogeneity would not necessarily affect the causal estimates. Another sensitivity analysis was performed for IVW analysis accounting for the LD structure of the instruments using the mr_ivw function in the MendelianRandomization package in R; these results were reported along with the IVW results in the main analysis. False discovery rate correction was applied to the P for the causal estimates of the remaining exposure-outcome pairs.

Supplementary Results

Baseline characteristics and metabolomic data in the NHS and HPFS

In NHS, 28.7% of the *APOE4* carriers had a family history of dementia in NHS (n =295). Participants with a high polygenic risk score (PRS) of Alzheimer's disease and related dementias (AD/ADRD), excluding the *APOE* region, had a more prevalent family history of dementia (highest tertile, 25.6%) compared to those with a low PRS of ADRD (lowest tertile, 19.9%). *APOE4* homozygotes and participants with a high PRS of ADRD, including the two *APOE* variants, had a high prevalence of history of hypercholesterolemia (50.8% for *APOE4* homozygotes and 34.1% for the highest tertile of ADRD PRS); no material differences in the prevalence was observed across tertiles of the ADRD PRS excluding the *APOE* region, supporting the role of *APOE4* in cholesterol dysregulation[11] (**Supplementary Table 1**). Similar patterns were observed in HPFS, where *APOE4* carriers had a higher prevalence of family history of dementia (22.0%) and hypercholesterolemia (48.5%) (**Supplementary Table 2**).

As expected, the majority of 401 metabolites in NHS were lipids and lipid-like molecules, while non-lipid metabolites accounted for 35.4% of the total (**Fig. 1b**). A subset of metabolites, such as trimethylamine N-oxide[12], allantoin[13], and piperine[14], has been previously associated with ADRD.

Validation of the dementia outcome against phosphorylated Tau 217 in NHS

As a validation of the dementia outcomes, we examined the association between plasma level of phosphorylated Tau 217 (p-Tau217), an established biomarker for early AD diagnosis[15], and the dementia endpoint in a subset of 103 NHS participants. These participants completed the Telephone Interviews for Cognitive Status between 1995 and 2008 and provided blood samples between 1989 and 1990. The subset comprised 51 individuals randomly selected from the lowest quartile and 52 from the highest quartile of global cognition scores. At the time of blood sample collection, all participants were free of cognitive impairment, Parkinson's disease, stroke, and cancer.

Plasma p-Tau217 levels were quantified using the ALZpath assay on a Simoa HD-X analyzer (Quanterix Corporation, Billerica, MA) at the Clinical and Translational Research Unit Biomarker Core, Massachusetts Alzheimer's Disease Research Center, and Massachusetts General Hospital. A split pilot study conducted on 14 plasma duplicate samples demonstrated the assay's excellent reproducibility, with a mean coefficient of variation of 6.3% in the NHS plasma samples.

Over a follow-up period of up to 34 years (1989–2023), 27 participants developed incident dementia. Cox PH models were applied to evaluate dementia risk, adjusting for age, family history of dementia, and history of depression or regular antidepressant use. Elevated plasma p-Tau217 levels were significantly associated with an increased risk of dementia, with a hazard ratio (HR; 95% confidence interval [CI]) of 2.78 (1.02, 7.52) comparing the highest to the lowest quartile of p-Tau217 levels (P for linear trend $=1.39\times10^{-4}$).

Validation of the genetic associations with cognitive outcomes in NHS and HPFS

As a validation of the genetic associations with cognitive outcomes in both NHS and HPFS, we examined associations between *APOE4* genotype, ADRD PRSs, the dementia endpoint, and cognitive function (NHS only). Cox PH models were fitted for the dementia outcome and generalized linear models were used for cognitive function outcomes, adjusting for age, the top 4 genetic principal components, and genotyping platforms. In NHS, we confirmed that carrying *APOE4* alleles or having a high genetic risk of ADRD (as represented by the PRSs) was associated with an increased risk of dementia (HR [95% CI] =2.16 [1.78, 2.60] for *APOE4* heterozygote and 5.37 [3.40, 8.48] for *APOE4* homozygote compared to noncarrier; HR [95% CI] =1.34 [1.22, 1.46] for one-unit increase in ADRD PRS, excluding *APOE* region; all with P <0.001) and poorer cognitive function (−0.17 in TICS score [95% CI: -0.28, −0.06] for *APOE4* carrier compared to noncarrier, P =0.003; −0.05 in TICS score [95% CI: −0.10, −0.002] for one-unit increase in ADRD PRS, excluding *APOE* region, P =0.04) in our study population (**Fig. 1d**, **Supplementary Fig. 1**, and **Supplementary Table 4**). Similarly in HPFS, carrying *APOE4* alleles or having a high ADRD PRS was associated with an increased risk of dementia (HR [95% CI] =6.29 [2.68, 14.73] for *APOE4* homozygote compared to noncarrier; HR [95% CI] =1.35 [1.32, 2.05] for one-unit increase in ADRD PRS, excluding *APOE* region; both with P <0.001) (**Extended Data Fig. 1b** and **Supplementary Table 4**).

Common AD/ADRD risk variants modified the associations between lipid metabolites and dementia risk

Lipid metabolites showed significant interactions with AD/ADRD genetic variants in relation to dementia risk. For example, C32:2 phosphatidylcholine, a phospholipid involved in maintaining membrane structure, fluidity, and signaling, exhibited a positive interaction with a variant (rs2830489-T) mapped to the *ADAMTS1* gene (**Fig. 2c**).[16] *ADAMTS1* has been implicated in neurodegenerative diseases due to its role in extracellular matrix (ECM) remodeling and neuroinflammation in the brain.[17] This positive interaction may arise from disrupted

phosphocholine metabolism, which could increase *ADAMTS1* activity indirectly through elevated lysophosphatidylcholine production[18], thereby promoting neuroinflammation and ECM alterations that compromise neuronal health.

MedDiet has the potential to exert a more pronounced modulatory effect on metabolites implicated in dementia risk in *APOE4* homozygotes

We identified nominally significant interactions between MedDiet and *APOE4* genotype in relation to 1,7-dimethyluric acid, a derivative of caffeine metabolism with established antioxidant properties and potential neuroprotective effects.[19,20] Protective associations were observed between 1,7-dimethyluric acid and dementia risk across all participants, as well as within *APOE4* subgroups in our study population, suggesting MedDiet's potential to influence 1,7-dimethyluric acid levels, in an *APOE4*-dependent way, which may in turn be linked to dementia risk. Another suggestive positive interaction was observed for MedDiet and *APOE4* homozygosity in relation to N-acetylornithine, a metabolite involved in arginine and proline metabolism, as well as the urea cycle.[21] Intriguingly, the increased level of N-acetylornithine was found to be nonsignificantly associated with elevated dementia risk only among *APOE4* homozygotes, among which the inverse association between MedDiet and N-acetylornithine was also more pronounced.

Putative causal relationships between metabolomic features and cognitive function

N6-carbamoylthreonyladenosine (t^6A) exhibited a strong beneficial effect on cognitive performance. t^6A is critical for maintaining the stability and function of tRNAs, allowing for proper codon recognition during the translation process in protein synthesis.[22-25] Impaired t^6A biosynthesis could cause oxidative stress and mitochondrial dysfunction[26,27], both implicated in neurodegeneration[24]. A number of causal relationships were identified between metabolites involved in adenosine triphosphate metabolism, including adenosine 5'-monophosphate, adenosine 5'-diphosphate, phosphate, and flavin adenine dinucleotide[28], and cognitive function, highlighting the role of energy imbalances, mitochondrial dysfunction, and impaired oxidative damage neutralization, which may exacerbate neuronal dysfunction and cognitive decline.[29,30]

Supplementary Discussion

While few metabolites identified in the two-sample MR analysis were also included in the NHS analysis, none showed significant associations with dementia risk in NHS (**Supplementary Tables 7** and **26**). This is expected, as MR leverages large-scale GWAS summary statistics to maximize power for detecting causal effects. In contrast, NHS provides individual-level, longitudinal data for modeling gene-metabolite interactions and revealing mechanisms relevant to prevention, but may not have comparable power to MR. This highlights the complementary strengths of the two approaches.

While omics-based prediction models show promise for risk stratification, real-world implementation remains challenging. Nonetheless, emerging clinical applications of PRS and other omics-based models, such as aging clocks, demonstrate translational potential.[31,32] As these technologies become more affordable and scalable, such approaches may become more practical for clinical use and precision prevention.

References

1. Zeleznik, O.A., *et al.* A Prospective Analysis of Circulating Plasma Metabolites Associated with Ovarian Cancer Risk. *Cancer Res* **80**, 1357-1367 (2020).
2. O'Sullivan, J.F., *et al.* Dimethylguanidino valeric acid is a marker of liver fat and predicts diabetes. *J Clin Invest* **127**, 4394-4402 (2017).
3. DeVille, N.V., *et al.* Neighborhood socioeconomic status and mortality in the nurses' health study (NHS) and the nurses' health study II (NHSII). *Environ Epidemiol* **7**, e235 (2023).
4. Wolf, A.M., *et al.* Reproducibility and validity of a self-administered physical activity questionnaire. *Int J Epidemiol* **23**, 991-999 (1994).
5. Chen, Y., *et al.* Genomic atlas of the plasma metabolome prioritizes metabolites implicated in human diseases. *Nat Genet* **55**, 44-53 (2023).
6. Yang, J., *et al.* Conditional and joint multiple-SNP analysis of GWAS summary statistics identifies additional variants influencing complex traits. *Nat Genet* **44**, 369-375, S361-363 (2012).
7. Hemani, G., *et al.* The MR-Base platform supports systematic causal inference across the human phenome. *Elife* **7**(2018).
8. Kurki, M.I., *et al.* FinnGen provides genetic insights from a well-phenotyped isolated population. *Nature* **613**, 508-518 (2023).
9. Lee, J.J., *et al.* Gene discovery and polygenic prediction from a genome-wide association study of educational attainment in 1.1 million individuals. *Nat Genet* **50**, 1112-1121 (2018).
10. Wightman, D.P., *et al.* A genome-wide association study with 1,126,563 individuals identifies new risk loci for Alzheimer's disease. *Nat Genet* **53**, 1276-1282 (2021).
11. Mahley, R.W. Central Nervous System Lipoproteins: ApoE and Regulation of Cholesterol Metabolism. *Arterioscler Thromb Vasc Biol* **36**, 1305-1315 (2016).
12. Vogt, N.M., *et al.* The gut microbiota-derived metabolite trimethylamine N-oxide is elevated in Alzheimer's disease. *Alzheimers Res Ther* **10**, 124 (2018).
13. Ahn, Y.J., *et al.* Effects of allantoin on cognitive function and hippocampal neurogenesis. *Food Chem Toxicol* **64**, 210-216 (2014).
14. Chonpathompikunlert, P., Wattanathorn, J. & Muchimapura, S. Piperine, the main alkaloid of Thai black pepper, protects against neurodegeneration and cognitive impairment in animal model of cognitive deficit like condition of Alzheimer's disease. *Food Chem Toxicol* **48**, 798-802 (2010).
15. Ashton, N.J., *et al.* Diagnostic Accuracy of a Plasma Phosphorylated Tau 217 Immunoassay for Alzheimer Disease Pathology. *JAMA Neurol* **81**, 255-263 (2024).
16. van der Veen, J.N., *et al.* The critical role of phosphatidylcholine and phosphatidylethanolamine metabolism in health and disease. *Biochim Biophys Acta Biomembr* **1859**, 1558-1572 (2017).
17. Gottschall, P.E. & Howell, M.D. ADAMTS expression and function in central nervous system injury and disorders. *Matrix Biol* **44-46**, 70-76 (2015).
18. Law, S.H., *et al.* An Updated Review of Lysophosphatidylcholine Metabolism in Human Diseases. *Int J Mol Sci* **20**(2019).
19. Fujimaki, M., *et al.* Serum caffeine and metabolites are reliable biomarkers of early Parkinson disease. *Neurology* **90**, e404-e411 (2018).
20. Haberman, F., *et al.* Soluble neuroprotective antioxidant uric acid analogs ameliorate ischemic brain injury in mice. *Neuromolecular Med* **9**, 315-323 (2007).
21. Zheng, H.K., *et al.* Metabolic reprogramming of the urea cycle pathway in experimental pulmonary arterial hypertension rats induced by monocrotaline. *Respir Res* **19**, 94 (2018).
22. Deutsch, C., El Yacoubi, B., de Crecy-Lagard, V. & Iwata-Reuyl, D. Biosynthesis of threonylcarbamoyl adenosine (t6A), a universal tRNA nucleoside. *J Biol Chem* **287**, 13666-13673 (2012).

23. Swinehart, W., *et al.* Specificity in the biosynthesis of the universal tRNA nucleoside N(6)-threonylcarbamoyl adenosine (t(6)A)-TsaD is the gatekeeper. *RNA* **26**, 1094-1103 (2020).
24. Edvardson, S., *et al.* tRNA N6-adenosine threonylcarbamoyltransferase defect due to KAE1/TCS3 (OSGEP) mutation manifest by neurodegeneration and renal tubulopathy. *Eur J Hum Genet* **25**, 545-551 (2017).
25. Su, C., Jin, M. & Zhang, W. Conservation and Diversification of tRNA t(6)A-Modifying Enzymes across the Three Domains of Life. *Int J Mol Sci* **23**(2022).
26. Zhang, Y., Zhou, J.B., Yin, Y., Wang, E.D. & Zhou, X.L. Multifaceted roles of t6A biogenesis in efficiency and fidelity of mitochondrial gene expression. *Nucleic Acids Res* **52**, 3213-3233 (2024).
27. Zhou, J.B., *et al.* Molecular basis for t6A modification in human mitochondria. *Nucleic Acids Res* **48**, 3181-3194 (2020).
28. Dunn, J. & Grider, M.H. Physiology, Adenosine Triphosphate. in *StatPearls* (Treasure Island (FL), 2024).
29. Liu, Y., Hyde, A.S., Simpson, M.A. & Barycki, J.J. Emerging regulatory paradigms in glutathione metabolism. *Adv Cancer Res* **122**, 69-101 (2014).
30. Sekhar, R.V., *et al.* Deficient synthesis of glutathione underlies oxidative stress in aging and can be corrected by dietary cysteine and glycine supplementation. *Am J Clin Nutr* **94**, 847-853 (2011).
31. Moqri, M., *et al.* Biomarkers of aging for the identification and evaluation of longevity interventions. *Cell* **186**, 3758-3775 (2023).
32. Mavaddat, N., *et al.* Polygenic Risk Scores for Prediction of Breast Cancer and Breast Cancer Subtypes. *Am J Hum Genet* **104**, 21-34 (2019).

Supplementary Figures

Supplementary Fig. 1: Cumulative incidence of dementia over time stratified by tertiles of ADRD PRS (including *APOE* variants) in NHS

Supplementary Fig. 2: Associations between metabolites and dementia risk for metabolites with significant interactions with *APOE4* genotype in relation to dementia risk in NHS (FDR <0.05)

Supplementary Fig. 3: Consistency of metabolite-*APOE4* interaction results from models adjusting for family history of dementia (primary analysis) and not adjusting for family history of dementia (sensitivity analysis) in NHS

Supplementary Fig. 4: Consistency of metabolite-*APOE4* interaction results across models with the composite dementia endpoint, self-reported dementia case, and dementia death as dependent variables in NHS

Supplementary Fig. 5: Associations between metabolites and dementia risk for metabolites with significant interactions with common AD/ADRD variants in relation to dementia risk in NHS (FDR <0.05)

Supplementary Fig. 6: Associations between the MedDiet index score and global cognition and verbal memory scores in NHS

Supplementary Fig. 7: Consistency of the associations between MedDiet adherence and plasma metabolites in NHS and HPFS

Supplementary Fig. 8: Prediction results for global cognition and verbal memory scores from random forest models in NHS

Supplementary Fig. 9: Regional genetic association plots of colocalized loci for metabolites or ratios and dementia or cognitive function

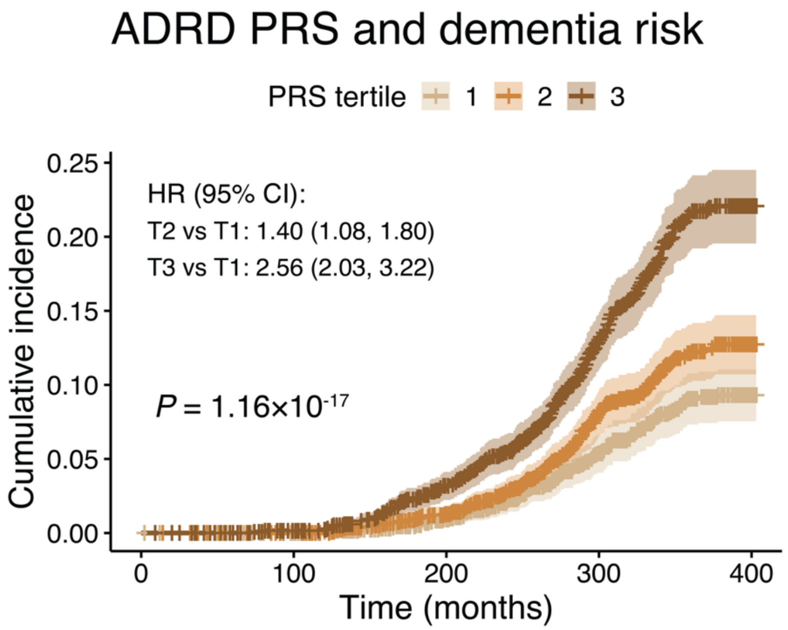

Supplementary Fig. 1: Cumulative incidence of dementia over time stratified by tertiles of ADRD PRS (including *APOE* variants) in NHS. Lines indicate cumulative incidence across *APOE4* genotypes and tertiles of polygenic risk score of ADRD (including *APOE* variants) over the follow-up period, with shaded areas representing 95% CIs. Consistent with the curves, unadjusted HRs were estimated using Cox PH models; covariate-adjusted HRs with 95% CIs are provided in Supplementary Table 4. Person-time was accrued from baseline until the earliest occurrence of an incident dementia case, dementia death, or the end of follow-up. The analyses were conducted among 4,215 NHS participants. The statistical test was two-sided.

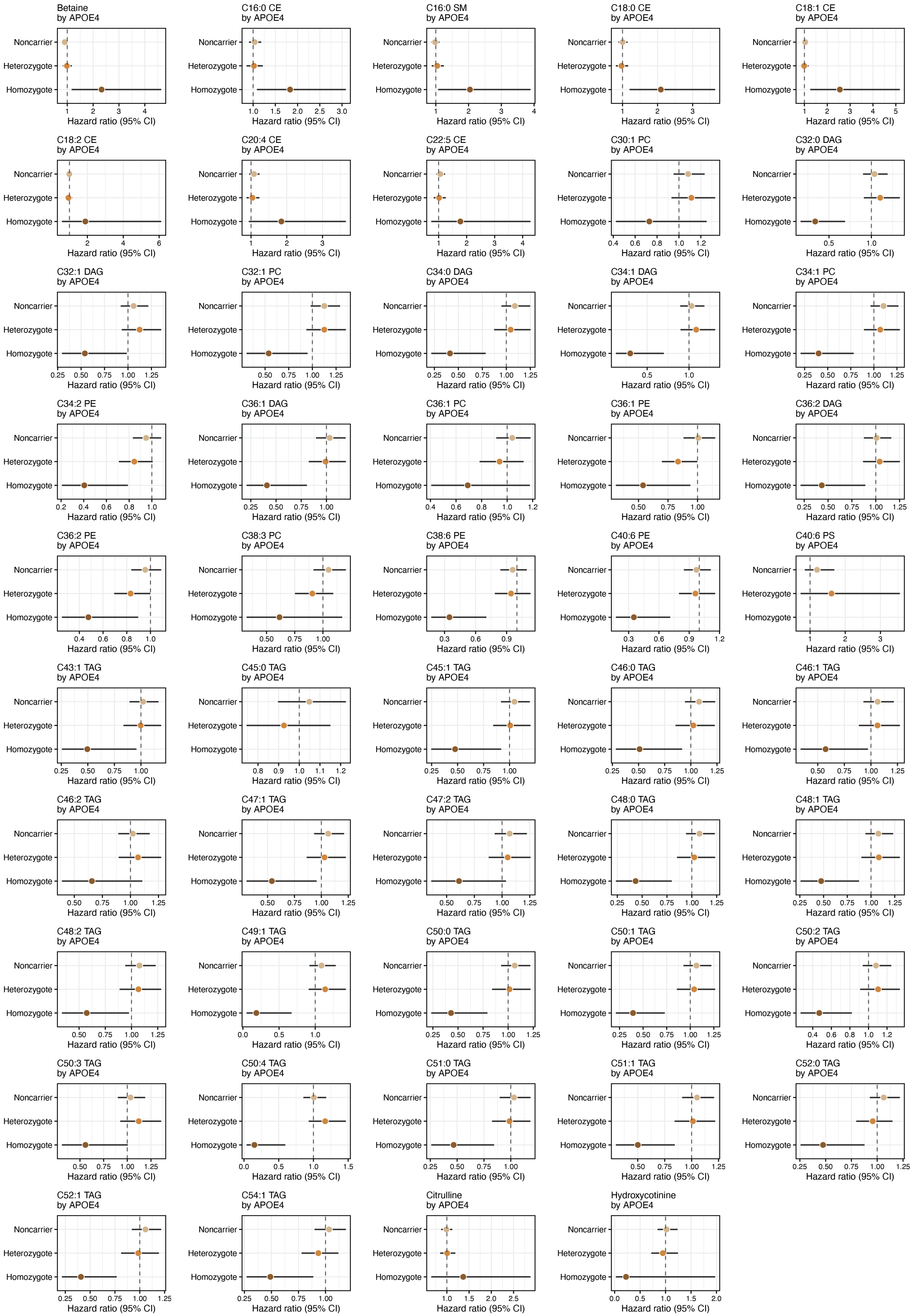
Betaine
by APOE4
Noncarrier
Heterozygote
Homozygote
1 2 3 4
Hazard ratio (95% CI)
C16:0 CE
by APOE4
Noncarrier
Heterozygote
Homozygote
1.0 1.5 2.0 2.5 3.0
Hazard ratio (95% CI)
C16:0 SM
by APOE4
Noncarrier
Heterozygote
Homozygote
1 2 3 4
Hazard ratio (95% CI)
C18:0 CE
by APOE4
Noncarrier
Heterozygote
Homozygote
1 2 3
Hazard ratio (95% CI)
C18:1 CE
by APOE4
Noncarrier
Heterozygote
Homozygote
1 2 3 4 5
Hazard ratio (95% CI)
C18:2 CE
by APOE4
Noncarrier
Heterozygote
Homozygote
2 4 6
Hazard ratio (95% CI)
C20:4 CE
by APOE4
Noncarrier
Heterozygote
Homozygote
1 2 3
Hazard ratio (95% CI)
C22:5 CE
by APOE4
Noncarrier
Heterozygote
Homozygote
1 2 3 4
Hazard ratio (95% CI)
C30:1 PC
by APOE4
Noncarrier
Heterozygote
Homozygote
0.4 0.6 0.8 1.0 1.2
Hazard ratio (95% CI)
C32:0 DAG
by APOE4
Noncarrier
Heterozygote
Homozygote
0.5 1.0
Hazard ratio (95% CI)
C32:1 DAG
by APOE4
Noncarrier
Heterozygote
Homozygote
0.25 0.50 0.75 1.00 1.25
Hazard ratio (95% CI)
C32:1 PC
by APOE4
Noncarrier
Heterozygote
Homozygote
0.50 0.75 1.00 1.25
Hazard ratio (95% CI)
C34:0 DAG
by APOE4
Noncarrier
Heterozygote
Homozygote
0.25 0.50 0.75 1.00 1.25
Hazard ratio (95% CI)
C34:1 DAG
by APOE4
Noncarrier
Heterozygote
Homozygote
0.5 1.0
Hazard ratio (95% CI)
C34:1 PC
by APOE4
Noncarrier
Heterozygote
Homozygote
0.25 0.50 0.75 1.00 1.25
Hazard ratio (95% CI)
C34:2 PE
by APOE4
Noncarrier
Heterozygote
Homozygote
0.2 0.4 0.6 0.8 1.0
Hazard ratio (95% CI)
C36:1 DAG
by APOE4
Noncarrier
Heterozygote
Homozygote
0.25 0.50 0.75 1.00
Hazard ratio (95% CI)
C36:1 PC
by APOE4
Noncarrier
Heterozygote
Homozygote
0.4 0.6 0.8 1.0 1.2
Hazard ratio (95% CI)
C36:1 PE
by APOE4
Noncarrier
Heterozygote
Homozygote
0.50 0.75 1.00
Hazard ratio (95% CI)
C36:2 DAG
by APOE4
Noncarrier
Heterozygote
Homozygote
0.25 0.50 0.75 1.00 1.25
Hazard ratio (95% CI)
C36:2 PE
by APOE4
Noncarrier
Heterozygote
Homozygote
0.4 0.6 0.8 1.0
Hazard ratio (95% CI)
C38:3 PC
by APOE4
Noncarrier
Heterozygote
Homozygote
0.50 0.75 1.00
Hazard ratio (95% CI)
C38:6 PE
by APOE4
Noncarrier
Heterozygote
Homozygote
0.3 0.6 0.9
Hazard ratio (95% CI)
C40:6 PE
by APOE4
Noncarrier
Heterozygote
Homozygote
0.3 0.6 0.9 1.2
Hazard ratio (95% CI)
C40:6 PS
by APOE4
Noncarrier
Heterozygote
Homozygote
1 2 3
Hazard ratio (95% CI)
C43:1 TAG
by APOE4
Noncarrier
Heterozygote
Homozygote
0.25 0.50 0.75 1.00
Hazard ratio (95% CI)
C45:0 TAG
by APOE4
Noncarrier
Heterozygote
Homozygote
0.8 0.9 1.0 1.1 1.2
Hazard ratio (95% CI)
C45:1 TAG
by APOE4
Noncarrier
Heterozygote
Homozygote
0.25 0.50 0.75 1.00
Hazard ratio (95% CI)
C46:0 TAG
by APOE4
Noncarrier
Heterozygote
Homozygote
0.25 0.50 0.75 1.00 1.25
Hazard ratio (95% CI)
C46:1 TAG
by APOE4
Noncarrier
Heterozygote
Homozygote
0.50 0.75 1.00 1.25
Hazard ratio (95% CI)
C46:2 TAG
by APOE4
Noncarrier
Heterozygote
Homozygote
0.50 0.75 1.00 1.25
Hazard ratio (95% CI)
C47:1 TAG
by APOE4
Noncarrier
Heterozygote
Homozygote
0.50 0.75 1.00 1.25
Hazard ratio (95% CI)
C47:2 TAG
by APOE4
Noncarrier
Heterozygote
Homozygote
0.50 0.75 1.00 1.25
Hazard ratio (95% CI)
C48:0 TAG
by APOE4
Noncarrier
Heterozygote
Homozygote
0.25 0.50 0.75 1.00 1.25
Hazard ratio (95% CI)
C48:1 TAG
by APOE4
Noncarrier
Heterozygote
Homozygote
0.25 0.50 0.75 1.00 1.25
Hazard ratio (95% CI)
C48:2 TAG
by APOE4
Noncarrier
Heterozygote
Homozygote
0.50 0.75 1.00 1.25
Hazard ratio (95% CI)
C49:1 TAG
by APOE4
Noncarrier
Heterozygote
Homozygote
0.0 0.5 1.0
Hazard ratio (95% CI)
C50:0 TAG
by APOE4
Noncarrier
Heterozygote
Homozygote
0.25 0.50 0.75 1.00 1.25
Hazard ratio (95% CI)
C50:1 TAG
by APOE4
Noncarrier
Heterozygote
Homozygote
0.25 0.50 0.75 1.00 1.25
Hazard ratio (95% CI)
C50:2 TAG
by APOE4
Noncarrier
Heterozygote
Homozygote
0.4 0.6 0.8 1.0 1.2
Hazard ratio (95% CI)
C50:3 TAG
by APOE4
Noncarrier
Heterozygote
Homozygote
0.50 0.75 1.00 1.25
Hazard ratio (95% CI)
C50:4 TAG
by APOE4
Noncarrier
Heterozygote
Homozygote
0.0 0.5 1.0 1.5
Hazard ratio (95% CI)
C51:0 TAG
by APOE4
Noncarrier
Heterozygote
Homozygote
0.25 0.50 0.75 1.00
Hazard ratio (95% CI)
C51:1 TAG
by APOE4
Noncarrier
Heterozygote
Homozygote
0.50 0.75 1.00 1.25
Hazard ratio (95% CI)
C52:0 TAG
by APOE4
Noncarrier
Heterozygote
Homozygote
0.25 0.50 0.75 1.00 1.25
Hazard ratio (95% CI)
C52:1 TAG
by APOE4
Noncarrier
Heterozygote
Homozygote
0.25 0.50 0.75 1.00 1.25
Hazard ratio (95% CI)
C54:1 TAG
by APOE4
Noncarrier
Heterozygote
Homozygote
0.25 0.50 0.75 1.00
Hazard ratio (95% CI)
Citrulline
by APOE4
Noncarrier
Heterozygote
Homozygote
1.0 1.5 2.0 2.5
Hazard ratio (95% CI)
Hydroxycotinine
by APOE4
Noncarrier
Heterozygote
Homozygote
0.0 0.5 1.0 1.5 2.0
Hazard ratio (95% CI)

Supplementary Fig. 2: Associations between metabolites and dementia risk for metabolites with significant interactions with *APOE4* genotype in relation to dementia risk in NHS (FDR <0.05). Stratified HRs and 95% CIs for dementia risk per one-SD increment in metabolite level, categorized by *APOE4* genotype are shown. Some stratified results are not shown due to data sparsity. The analyses were conducted among 4,215 NHS participants. All statistical tests were two-sided.

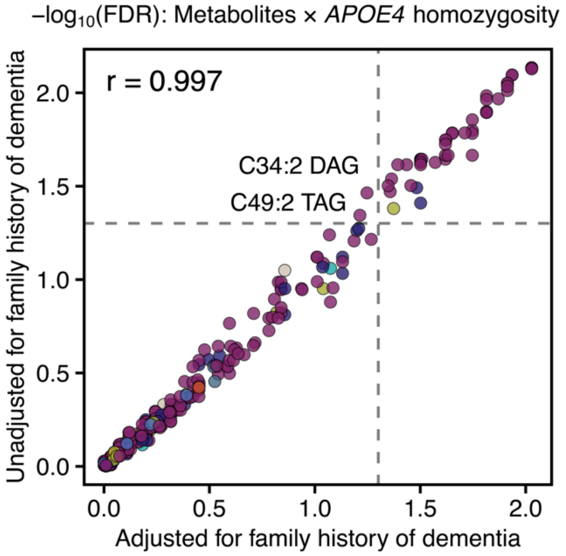

Supplementary Fig. 3: Consistency of metabolite-*APOE4* interaction results from models adjusting for family history of dementia (primary analysis) and not adjusting for family history of dementia (sensitivity analysis) in NHS. Each dot represents a metabolite, colored by HMDB superclass (see Fig. 2 for legend). The Pearson correlation coefficient between the -$\log_{10}$(FDR) for interaction terms between metabolites and *APOE4* homozygosity estimated from Cox PH models from the adjusted and unadjusted models is annotated on the figure. The analyses were conducted among 4,215 NHS participants. All statistical tests were two-sided.

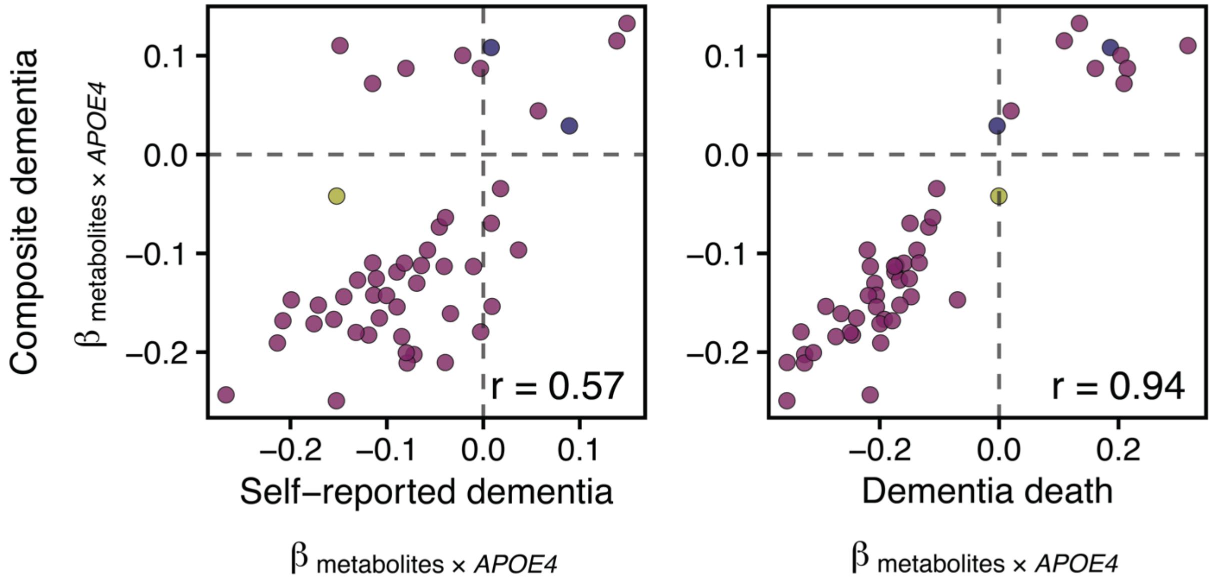

Supplementary Fig. 4: Consistency of metabolite-*APOE4* interaction results across models with the composite dementia endpoint, self-reported dementia case, and dementia death as dependent variables in NHS. Each dot represents a metabolite with significant *APOE4* interactions, colored by the HMDB superclass (see Fig. 2 for legend). Pearson correlation coefficients in the beta coefficients for interaction terms between metabolites and *APOE4* carrier status estimated from Cox PH models with the composite dementia endpoint, self-reported dementia case, and dementia death as the dependent variable are annotated on each figure. The analyses were conducted among 4,215 NHS participants. All statistical tests were two-sided.

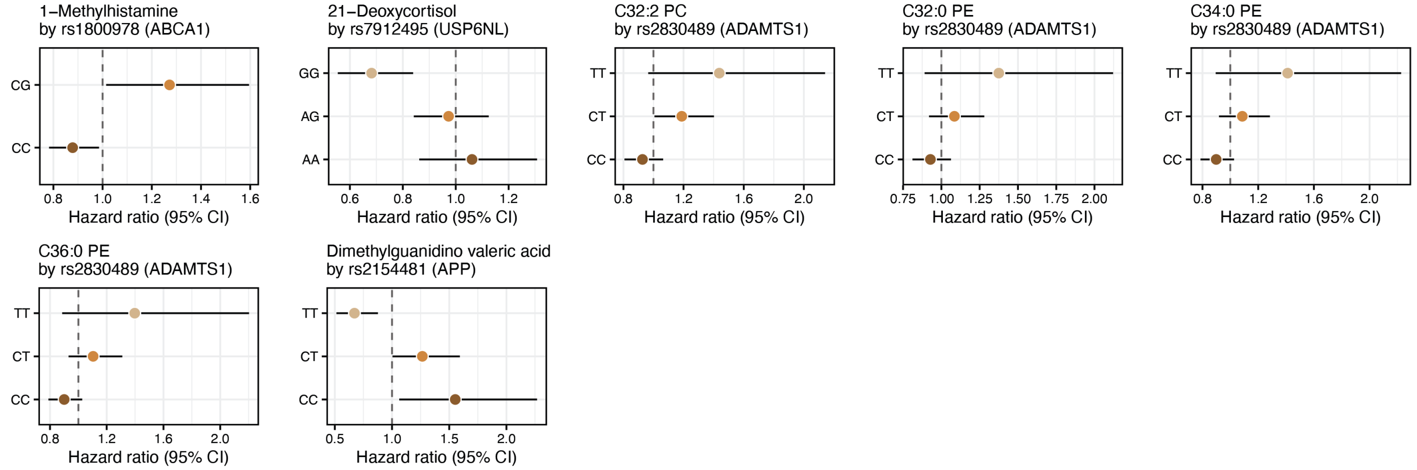

Supplementary Fig. 5: Associations between metabolites and dementia risk for metabolites with significant interactions with common AD/ADRD variants in relation to dementia risk in NHS (FDR <0.05). Stratified HRs and 95% CIs for dementia risk per one-SD increment in metabolite level, categorized by genotype groups are shown. Genotype groups were defined based on rounded allele dosages. Some stratified results are not shown due to data sparsity. The analyses were conducted among 4,215 NHS participants. All statistical tests were two-sided.

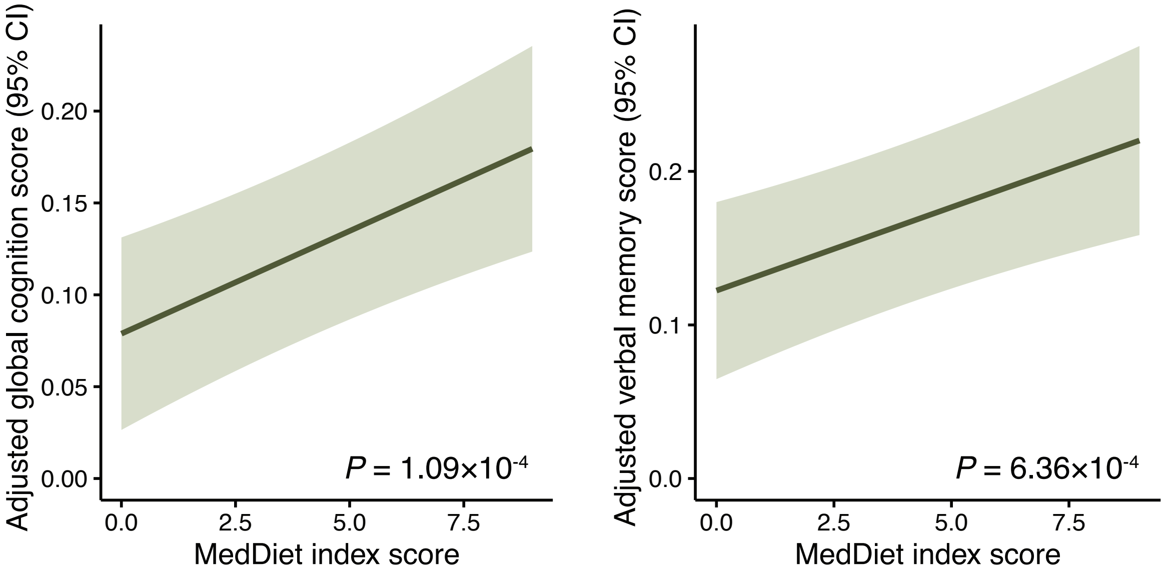

Supplementary Fig. 6: Associations between the MedDiet index score and global cognition and verbal memory scores in NHS. Generalized linear models estimated the global cognition and verbal memory scores and corresponding 95% CIs across different MedDiet index levels. The analyses were conducted among NHS participants with cognitive and dietary data (n =16,244). All statistical tests were two-sided.

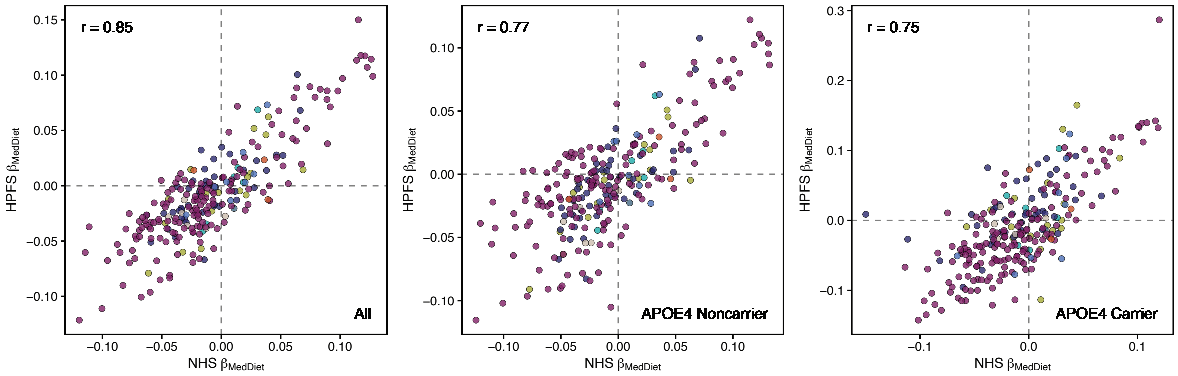

Supplementary Fig. 7: Consistency of the associations between MedDiet adherence and plasma metabolites in NHS and HPFS. Each dot represents a metabolite, colored by HMDB superclass (n =254 available in both cohorts; see Fig. 2 for legend). Pearson correlation coefficients of the beta estimates for the MedDiet index score in the full data and by *APOE4* carrier status, estimated from generalized linear models with metabolites as the dependent variable in NHS and HPFS, are annotated on the figure. *APOE4* carriers were not further divided into heterozygotes and homozygotes due to data sparsity among homozygotes with non-missing values for each metabolite in HPFS. The analyses were conducted among 4,215 NHS and 1,490 HPFS participants.

	**Global cognition** (AUC)			**Verbal memory** (AUC)		
	**All**	***APOE4* carrier**	***APOE4* noncarrier**	**All**	***APOE4* carrier**	***APOE4* noncarrier**
Baseline model	0.63	0.59	0.67	0.59	0.67	0.63
+ *APOE4*	0.62	/	/	0.59	/	/
+ *APOE4* + PRS	0.62	0.56	0.67	0.57	0.66	0.62
+ *APOE4* + PRS + Metabolites	0.62	0.59	0.64	0.58	0.54	0.60

Supplementary Fig. 8: Prediction results for global cognition and verbal memory scores from random forest models in NHS. The heatmap displays AUCs from random forest models classifying participants in the highest versus lowest tertile of the global cognition and verbal memory scores. In subgroup analyses by *APOE4* carrier status, *APOE4* genotype was excluded as a predictor. For all analyses, the NHS dataset (n =4,215) was randomly divided into training (60%) and test (40%) sets; models were fitted on the training set and evaluated on the test set. All results shown are from the test set.

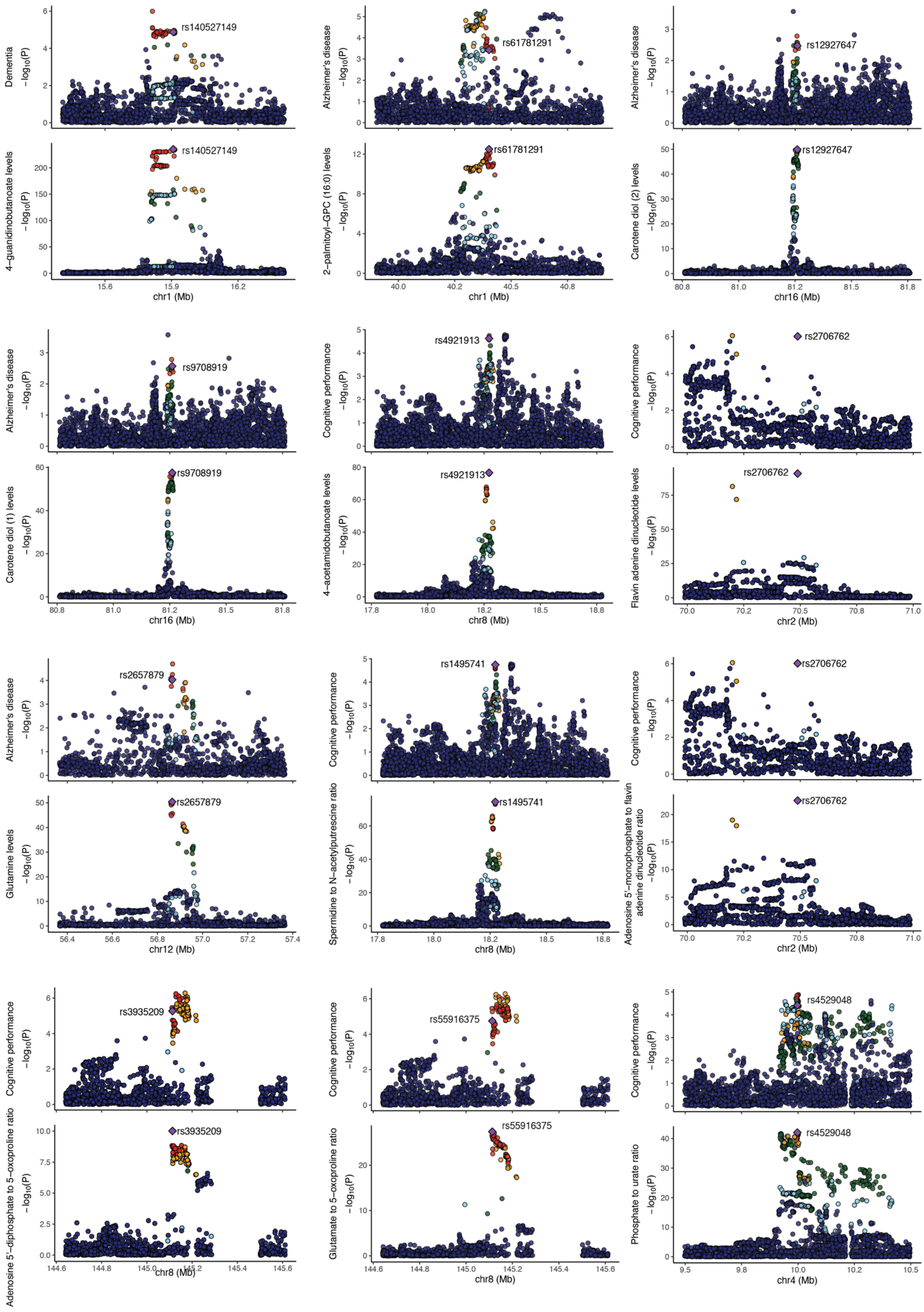
Dementia
rs140527149
4−guanidinobutanoate levels
chr1 (Mb)
Alzheimer's disease
rs61781291
2−palmitoyl−GPC (16:0) levels
rs12927647
Carotene diol (2) levels
chr16 (Mb)
rs9708919
Carotene diol (1) levels
Cognitive performance
rs4921913
4−acetamidobutanoate levels
chr8 (Mb)
rs2706762
Flavin adenine dinucleotide levels
chr2 (Mb)
rs2657879
Glutamine levels
chr12 (Mb)
rs1495741
Spermidine to N−acetylputrescine ratio
Adenosine 5'−monophosphate to flavin adenine dinucleotide ratio
rs3935209
Adenosine 5'−diphosphate to 5−oxoproline ratio
rs55916375
Glutamate to 5−oxoproline ratio
rs4529048
Phosphate to urate ratio
chr4 (Mb)
−log10(P)

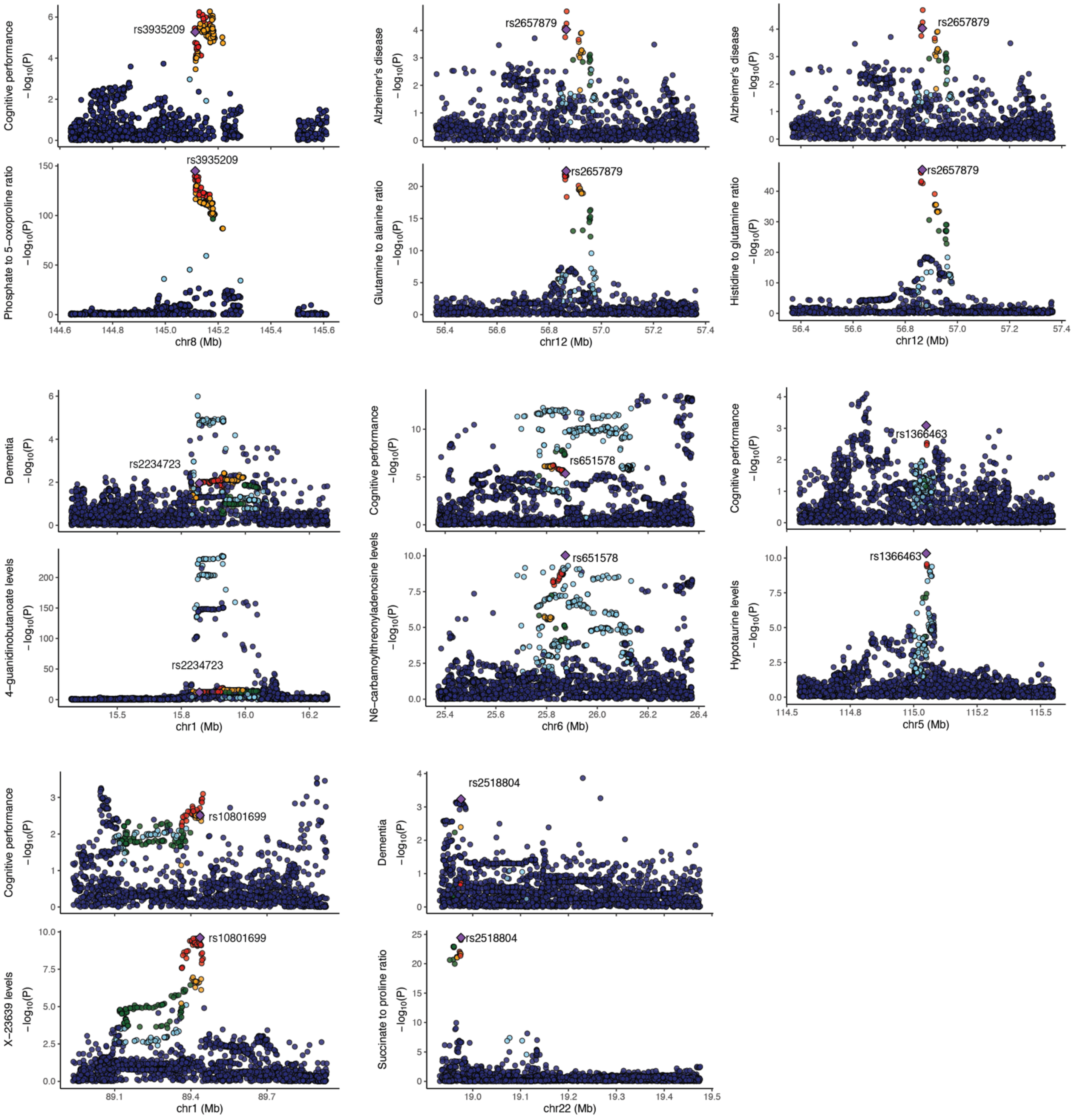

Supplementary Fig. 9: Regional genetic association plots of colocalized loci for metabolites or ratios and dementia or cognitive function. The plots display genetic association results for metabolites or ratios and dementia or cognitive function at colocalized loci with PP4/(PP3 + PP4) >70%. Each plot is annotated with the genetic instrument, and dots are color-coded according to their linkage disequilibrium with the instrumental variant. $-\log_{10}(P)$ for both metabolites or ratios and dementia or cognitive function were obtained from the original GWASs (Methods). The sample sizes for the original GWASs are as follows: metabolites or ratios (n = 8,299), cognitive performance (n = 257,841), dementia (5,933 cases / 166,584 controls), Alzheimer's disease (90,338 cases / 1,036,225 controls), and vascular dementia (881 cases / 211,508 controls) (Methods). All statistical tests were two-sided.
