# Supplementary material for: Interplay of genetic predisposition, plasma metabolome and Mediterranean diet in dementia risk and cognitive function
Source: Nat Med. 2025 Aug 25;31(11):3790–800. doi: 10.1038/s41591-025-03891-5 (PMC12618253; doi:10.1038/s41591-025-03891-5)
Supplement: Supplementary file 2 — Reporting Summary [file 41591_2025_3891_MOESM2_ESM.pdf]

nature portfolio

Corresponding author(s): Dong D. Wang

Last updated by author(s): Jun 20, 2025

Reporting Summary

Nature Portfolio wishes to improve the reproducibility of the work that we publish. This form provides structure for consistency and transparency in reporting. For further information on Nature Portfolio policies, see our Editorial Policies and the Editorial Policy Checklist.

Statistics

For all statistical analyses, confirm that the following items are present in the figure legend, table legend, main text, or Methods section.

n/a	Confirmed	
☐	☒	The exact sample size (*n*) for each experimental group/condition, given as a discrete number and unit of measurement
☐	☒	A statement on whether measurements were taken from distinct samples or whether the same sample was measured repeatedly
☐	☒	The statistical test(s) used AND whether they are one- or two-sided<br>*Only common tests should be described solely by name; describe more complex techniques in the Methods section.*
☐	☒	A description of all covariates tested
☐	☒	A description of any assumptions or corrections, such as tests of normality and adjustment for multiple comparisons
☐	☒	A full description of the statistical parameters including central tendency (e.g. means) or other basic estimates (e.g. regression coefficient) AND variation (e.g. standard deviation) or associated estimates of uncertainty (e.g. confidence intervals)
☐	☒	For null hypothesis testing, the test statistic (e.g. *F*, *t*, *r*) with confidence intervals, effect sizes, degrees of freedom and *P* value noted<br>*Give P values as exact values whenever suitable.*
☐	☒	For Bayesian analysis, information on the choice of priors and Markov chain Monte Carlo settings
☐	☒	For hierarchical and complex designs, identification of the appropriate level for tests and full reporting of outcomes
☐	☒	Estimates of effect sizes (e.g. Cohen's *d*, Pearson's *r*), indicating how they were calculated

Our web collection on statistics for biologists contains articles on many of the points above.

Software and code

Policy information about availability of computer code

Data collection	No software was used for data collection.
Data analysis	Genetic data were processed using PLINK v1.9<br>All data analyses and figure generation were conducted using R v4.2.0 (Main package: survival v3.7-0, caret v6.0-94, randomForest v4.7-1.2, TwoSampleMR v0.6.1, coloc v5.2.3, ggplot2 v3.5.0)<br>Analysis-specific programs are publicly available at https://github.com/DW-Group/Gene_Metabolites_MedDiet_Dementia

For manuscripts utilizing custom algorithms or software that are central to the research but not yet described in published literature, software must be made available to editors and reviewers. We strongly encourage code deposition in a community repository (e.g. GitHub). See the Nature Portfolio guidelines for submitting code & software for further information.

Data

Policy information about availability of data

All manuscripts must include a data availability statement. This statement should provide the following information, where applicable:

- Accession codes, unique identifiers, or web links for publicly available datasets
- A description of any restrictions on data availability
- For clinical datasets or third party data, please ensure that the statement adheres to our policy

Due to the gaining of informed consent from the participants, all the individual-level data from the Nurses' Health Study (NHS) and Health Professionals Follow-Up

Study (HPFS) are available through a request for external collaboration and upon approval of a letter of intent and a research proposal. Details on how to request external collaborations with the NHS can be found at https://nurseshealthstudy.org/researchers (contact principal investigator: Dr. A. Heather Eliassen, email: nhahe@channing.harvard.edu), and with HPFS at https://sites.sph.harvard.edu/hpfs/for-collaborators/ (contact principal investigator: Dr. Lorelei Mucci, email: lmucci@hsph.harvard.edu). external collaboration and upon approval of a letter of intent and a research proposal. Details on how to request external collaborations with the NHS can be found at https://nurseshealthstudy.org/researchers (contact principal investigator: A. H. Eliassen, email: nhahe@channing.harvard.edu).

Research involving human participants, their data, or biological material

Policy information about studies with human participants or human data. See also policy information about sex, gender (identity/presentation), and sexual orientation and race, ethnicity and racism.

Reporting on sex and gender	All NHS participants are female; all HPFS participants (replication cohort) are male.
Reporting on race, ethnicity, or other socially relevant groupings	The study population (NHS) consists predominantly of White, non-Hispanic females from 11 states in the United States; the replication cohort (HPFS) consists predominantly of White, non-Hispanic males from across the United States.
Population characteristics	The study population (NHS) included females who were under 75 years old, and free from dementia, Parkinson's disease, stroke, and cancer at baseline (1989-1992) when the blood samples were collected; the replication cohort (HPFS) included males who were under 75 years old, and free from dementia, Parkinson's disease, stroke, and cancer at baseline (1993-1996) when the blood samples were collected.
Recruitment	The NHS recruited female registered nurses from 11 states in the United States in 1976; the HPFS recruited male health professionals across the United States in 1986.
Ethics oversight	This study included de-identified data from participants who had consented to the use of their anonymized information for research purposes. Participants were not financially compensated for their participation. Approval for the study protocol of the Nurses' Health Study (NHS) and the Health Professionals Follow-Up Study (HPFS) were granted by the institutional review boards (IRB) of Brigham and Women's Hospital and the Harvard T.H. Chan School of Public Health (IRB protocol no. 1999P011114/BWH for NHS and IRB protocol no. HSPH 22067-102 for HPFS).

Note that full information on the approval of the study protocol must also be provided in the manuscript.

Field-specific reporting

Please select the one below that is the best fit for your research. If you are not sure, read the appropriate sections before making your selection.

☒ Life sciences ☐ Behavioural & social sciences ☐ Ecological, evolutionary & environmental sciences

For a reference copy of the document with all sections, see nature.com/documents/nr-reporting-summary-flat.pdf

Life sciences study design

All studies must disclose on these points even when the disclosure is negative.

Sample size	We included 4,215 females and 1,490 males who were under 75 years old, and free from dementia, Parkinson's disease, stroke, and cancer at baseline when the blood samples were collected; the blood was assayed for genetics and metabolomics.
Data exclusions	We excluded participants over 75 years old and those diagnosed with dementia, Parkinson's disease, stroke, or cancer at baseline, and those who did not provide blood samples for genetic and metabolomic profiling.
Replication	HPFS (1,490 males) was included as a replication cohort for findings from NHS (4,215 females).
Randomization	Observational study. No randomization.
Blinding	Observational study. No blinding.

Reporting for specific materials, systems and methods

We require information from authors about some types of materials, experimental systems and methods used in many studies. Here, indicate whether each material, system or method listed is relevant to your study. If you are not sure if a list item applies to your research, read the appropriate section before selecting a response.

Materials & experimental systems

n/a	Involved in the study
☒	☐ Antibodies
☒	☐ Eukaryotic cell lines
☒	☐ Palaeontology and archaeology
☒	☐ Animals and other organisms
☒	☐ Clinical data
☒	☐ Dual use research of concern
☒	☐ Plants

Methods

n/a	Involved in the study
☒	☐ ChIP-seq
☒	☐ Flow cytometry
☒	☐ MRI-based neuroimaging

Plants

Seed stocks	Not applicable.
Novel plant genotypes	Not applicable.
Authentication	Not applicable.
